# Modification of messenger RNA by 2′-*O*-methylation regulates gene expression in vivo

**DOI:** 10.1038/s41467-019-11375-7

**Published:** 2019-07-30

**Authors:** Brittany A. Elliott, Hsiang-Ting Ho, Srivathsan V. Ranganathan, Sweta Vangaveti, Olga Ilkayeva, Hala Abou Assi, Alex K. Choi, Paul F. Agris, Christopher L. Holley

**Affiliations:** 1 Department of Medicine, Duke University Medical Center, Durham, NC 27705 USA; 2 The RNA Institute, State University of New York, Albany, NY 12222 USA; 3 Duke Molecular Physiology Institute, Duke University, Durham, NC 27701 USA

**Keywords:** Small RNAs, RNA modification

## Abstract

Epitranscriptomic modifications of mRNA are important regulators of gene expression. While internal 2′-*O*-methylation (Nm) has been discovered on mRNA, questions remain about its origin and function in cells and organisms. Here, we show that internal Nm modification can be guided by small nucleolar RNAs (snoRNAs), and that these Nm sites can regulate mRNA and protein expression. Specifically, two box C/D snoRNAs (SNORDs) and the 2′-*O*-methyltransferase fibrillarin lead to Nm modification in the protein-coding region of *peroxidasin* (*Pxdn*). The presence of Nm modification increases *Pxdn* mRNA expression but inhibits its translation, regulating PXDN protein expression and enzyme activity both in vitro and in vivo. Our findings support a model in which snoRNA-guided Nm modifications of mRNA can regulate physiologic gene expression by altering mRNA levels and tuning protein translation.

## Abstract (web summary)

The post-transcriptional modification of mRNAs provides an additional layer to gene expression regulation. Here the authors show that 2′-*O*-methylation mediated by box C/D snoRNAs and fibrillarin can inhibit the translation of target mRNAs.

## Introduction

Post-transcriptional modifications of messenger RNA (mRNA) are emerging as an important layer of regulatory control over gene expression. Base modifications, especially *N*^6^-methyladenosine (m^6^A), influence mRNA splicing, stability, and translation by altering base-pair interactions and recruiting or inhibiting RNA-binding proteins^1–4^. While there is evidence that internal ribose modification by 2′-*O*-methylation (Nm) also occurs on mRNA, questions have been raised regarding its detection, and no biological role has been validated for this modification in cells or tissues^5,6^. Here we show that internal 2′-*O*-methylation of mRNA serves as a new mechanism of genetic regulatory control, with the ability to influence mRNA abundance and protein levels both in vitro and in vivo. Using genetic models, we show that the coding sequence of *peroxidasin* (*Pxdn*) mRNA undergoes Nm modification, and that this requires: (1) small nucleolar RNAs (snoRNAs), and (2) the snoRNA-guided 2′-*O*-methylase (*fibrillarin, Fbl*). In the case of *Pxdn*, Nm increases mRNA levels but inhibits protein synthesis. Our findings support the growing evidence that Nm modification of a codon can inhibit ribosomal protein translation by sterically blocking interactions between rRNA and the ribose backbone of the mRNA–tRNA minihelix^7–9^. Since there are hundreds of box C/D snoRNAs (SNORDs) that can guide Nm modifications, snoRNA-guided modification of mRNA could be a widespread mechanism of post-transcriptional gene regulation. Overall, these findings show that SNORDs guide 2′-*O*-methylation of mRNA, and that Nm can provide a significant post-transcriptional regulatory mechanism to regulate physiologic gene expression in vivo.

## Results

### Fibrillarin is required for 2′-*O*-methylation of Pxdn mRNA

**Fig. 1:**
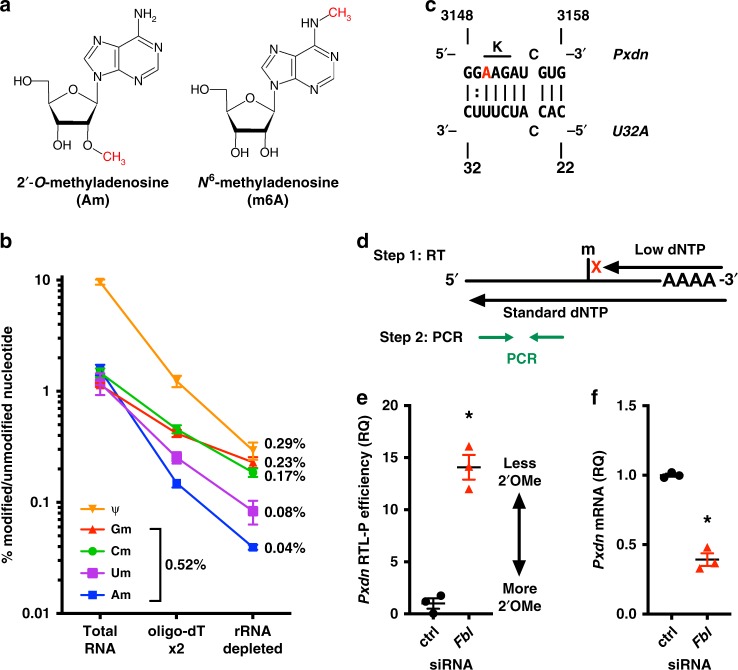
2′-*O*-methylation on *Pxdn* mRNA requires fibrillarin. **a** 2′-*O*-methylation (Nm) can occur in combination with any base. Nucleoside Am is shown, compared to base-modified m6A. **b** UPLC-MS/MS quantification of modified nucleosides on total RNA, mRNA after two rounds of oligo-dT selection, and mRNA further purified by rRNA depletion. **c** Predicted interaction between human snoRNA *U32A* (*U32A*) and peroxidasin (*Pxdn*). A3150 is the predicted Nm target (red), which is the first position of a lysine codon (K). **d** Illustration of RTL-P method. The amount of qPCR product from the low dNTP reactions of two different samples can be compared to establish the relative “RTL-P efficiency (RQ)”. RQ = relative quantity. See Methods for detail. **e**–**f** *Fbl* knockdown (KD) in HeLa cells, vs. negative control (ctrl) using siRNA for 48 h. *n* = 3 independent experiments, SEM error bars, *p < 0.05 by unpaired *t*-test. **e** *Fbl* KD leads to loss of Nm on *Pxdn* mRNA (*p* = 0.0005). **f** *Fbl* KD reduces *Pxdn* mRNA levels, normalized to *Rplp0* control transcript (*p* = 0.0002)

Nm modifications (Fig. 1a) are abundant on ribosomal RNA (rRNA), where they contribute to fine-tuning of ribosomal function^10^. These site-specific modifications are catalyzed by the enzyme FBL and guided by SNORDs via antisense complementarity. The primary role of SNORDs and FBL is to modify rRNA; however, recent findings suggest that some SNORDs have other functions^11–17^. In particular, four SNORDs from the *Rpl13a* genetic locus (*U32A*, *U33*, *U34*, and *U35A*) have an unexpected role regulating reactive oxygen species (ROS) and oxidative stress^18^. Loss of *Rpl13a* snoRNAs protects animals in models of sepsis and diabetes, but the mechanism for this phenotype has remained elusive, since it does not depend on rRNA modification^18–20^.

We therefore hypothesized that *Rpl13a* snoRNAs regulate ROS and oxidative stress by guiding Nm modification of mRNA, in addition to their roles modifying rRNA. Historically, mRNA Nm sites have been attributed to the 5′-cap, with an estimated abundance of around 0.2% (two modifications per transcript)^21,22^. To accurately measure the Nm content of highly purified mRNA using modern methods, we used ultra-high-performance liquid chromatography and tandem mass spectrometry (UPLC-MS/MS) to assay highly purified mouse liver mRNA. Pseudouridine content was a control for mRNA purity; we measured 0.3%, which is consistent with modern results^23^. No nucleosides were detected in mock samples lacking RNA (buffers and enzymes only). Measured Nm content was 0.5%, suggesting Nm sites are present on mRNA beyond the 5′-cap (Fig. 1b).

If mRNA 2′-*O*-methylation is guided by *Rpl13a* snoRNAs, the enzyme FBL must simultaneously interact with both the snoRNA and mRNA. We reasoned that the components of this complex might be captured by crosslinking and immunoprecipitation, and identified by RNA sequencing (CLIP-seq). Using published CLIP-seq data that captured RNAs in complex with the 2′-*O*-methylase FBL, we identified *Pxdn* mRNA as interacting with *Rpl13a* snoRNA *U32A*^24^. Like other peroxidases, PXDN consumes hydrogen peroxide to catalyze the production of hypohalous acids. This makes it an attractive candidate to help explain how the *Rpl13a* snoRNAs might influence ROS and oxidative stress.

SNORDs are characterized by box C and D consensus sequences, and they recognize RNA targets through antisense elements immediately 5′ to the D-box sites. The D’-box antisense element of human *U32A* (*hU32A*) has strong antisense complementarity to a highly conserved coding region of human *Pxdn* mRNA (Fig. 1c). Both *U32A* and *Pxdn* mRNA sequences are conserved in fly, mouse, and human (Supplementary Fig. 1a, b, c). Of note, *U51* redundantly targets the same 28S rRNA site as *U32A*, with nearly identical antisense elements to *U32A* across human, mouse, and fly (Supplementary Fig. 1c). As such, we considered both *U32A* and *U51* as candidates for guiding Nm-modification of *Pxdn* mRNA.

We thus tested whether the 2′-*O*-methylase *Fbl* is necessary for Nm modification of *Pxdn* mRNA. After knocking down *Fbl* in HeLa cells (Supplementary Fig. 1d, e), we assayed for Nm modification using the method of reverse transcription at low dNTP concentrations followed by PCR (RTL-P)^25^. This method provides a semi-quantitative assessment of Nm modification between two RNA samples by comparing the efficiency of PCR after reverse transcription (RT) reactions in low and normal dNTP concentrations. RT is inhibited at Nm sites under low dNTP conditions, but not at standard conditions, and the efficiency of the RT reaction is quantified by real-time PCR (Fig. 1d). Nm sites reduce RTL-P efficiency, and loss of Nm increases the RTL-P efficiency. *Fbl* knockdown led to increased RTL-P efficiency for *Pxdn* mRNA, consistent with loss of one or more snoRNA-guided Nm modifications (Fig. 1e). *Fbl* knockdown also lowered *Pxdn* mRNA expression by ~50% (Fig. 1f). Since Nm groups are added post-transcriptionally, this suggests that Nm modification may influence *Pxdn* mRNA stability.

### Box C/D snoRNAs are required for *Pxdn* mRNA Nm modification

**Fig. 2:**
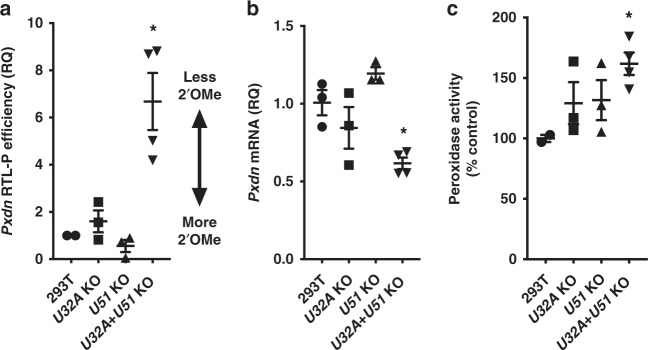
*Pxdn* mRNA modification by Nm requires box C/D snoRNAs. 293T cell lines with CRISPR/*Cas9* knockout (KO) for *U32A* (*n* = 3), *U51* (*n* = 3), or *U32A* + *U51* (*n* = 4). Each KO cell line is an independent biological sample, analyzed in two independent experiments. Mean and SEM error bars, *p < 0.05 vs. 293T, by one-way ANOVA. **a** *Pxdn* mRNA from snoRNA KO cells showed loss of Nm (*p = 0.0086). **b** snoRNA KO cells showed reduced *Pxdn* mRNA (*p = 0.0128). **c** Peroxidase activity in cells was measured using Amplex Red and normalized to WT (*p = 0.039)

Next, we tested whether snoRNAs *U32A* and *U51* are necessary for the Nm modification of *Pxdn* mRNA, by generating *U32A, U51*, and *U32A* + *U51* KO (snoKO) cell lines (validated in Supplementary Fig. 2a). Using RTL-P efficiency as a measure of Nm modification, only *U32A* + *U51* KO showed a significant loss of Nm on *Pxdn* mRNA (Fig. 2a). As an additional control, we tested two unrelated transcripts (*Gapdh* and *Pkm*) and found no difference in their relative RTL-P efficiency between WT and *U32A* + *U51* KO cells (Supplementary Fig. 2b, c). Together, these data suggest that *U32A* and *U51* redundantly target *Pxdn* mRNA for Nm modification at one or more sites. Loss of *U32A* + *U51* (but neither snoRNA alone) also resulted in significantly lower *Pxdn* mRNA levels (Fig. 2b), recapitulating the effect we saw with *Fbl* knockdown. In contrast, expression of control transcripts *Gapdh* and *Pkm* were unaffected in *U32A* + *U51* KO cells (Supplementary Fig. 2d). We initially hypothesized that this lower level of *Pxdn* mRNA expression would correspond to reduced peroxidase activity. However, we found that loss of *U32A* *+* *U51* caused a significant increase in peroxidase activity, while the loss of *U32A* or *U51* alone did not (Fig. 2c). These paradoxical results suggested that PXDN protein expression might be increased in response to loss of snoRNA-guided Nm modification on *Pxdn* mRNA.

### Regulation of *Pxdn* expression in vivo

*Pxdn* mRNA is expressed in most tissues, but PXDN protein (also known as vascular peroxidase 1, VPO1) has been found most abundantly in the heart, vasculature, and circulation^26,27^. Immunoblotting failed to detect significant amounts of PXDN protein in our 293T cells, despite robust mRNA expression and measurable peroxidase activity. We therefore analyzed hearts from mice that have been engineered for germline loss of all four *Rpl13a* snoRNAs, including *U32A* but not *U51* (sno −/–)^19^. The sno −/− mice have been previously validated and characterized, demonstrating reduced levels of ROS and oxidative stress in the pancreatic beta cells, and resistance to developing of diabetes^19^. Since prior studies have shown that haploinsufficiency of the *Rpl13a* snoRNAs results in lower levels of ROS and oxidative stress^18^, we tested both heterozygous (sno + /−) and homozygous (sno −/−) animals for relevant *Pxdn* phenotypes in the heart.

**Fig. 3:**
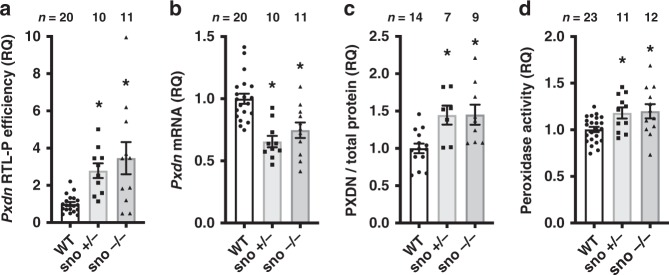
snoRNA-guided 2′-*O*-methylation regulates *Pxdn* mRNA expression, protein levels, and peroxidase activity in vivo. **a**–**d** Hearts from WT, sno +/−, and sno −/− mice were analyzed. Plots show mean ± SEM, normalized to WT control as relative quantities (RQ). *p < 0.05 vs. WT by one-way ANOVA. **a** *Pxdn* mRNA had less Nm in both sno +/− (*p = 0.021) and sno −/− (*p = 0.0007), vs. WT. **b** *Pxdn* mRNA abundance was reduced in sno +/− (*p = < 0.001) and sno −/− (*p = 0.0019) mice, vs. WT. **c** PXDN protein expression was higher in sno +/− (*p = 0.0163) and sno −/− (*p = 0.0079) mice, vs. WT (by immunoblotting with normalization to total protein per lane). **d** Peroxidase activity was higher in sno +/− (*p = 0.0447) and sno −/− mice (*p = 0.0177) vs. WT, as measured using Amplex Red

Results from these animals recapitulated our findings from cultured cells. Compared to WT hearts, both sno + /− and sno −/− hearts showed higher RTL-P efficiency for *Pxdn* mRNA, consistent with in vivo loss of Nm modification (Fig. 3a). Hearts from sno + /− and sno −/− mice also had lower expression of *Pxdn* mRNA (Fig. 3b). As predicted by our cell culture model, PXDN protein was elevated in both sno + /− and sno −/− hearts (Fig. 3c), and cardiac peroxidase activity was correspondingly increased in both the sno + /− and sno −/− animals (Fig. 3d). Although this genetic model is unable to specifically implicate loss of *U32A* as the sole reason for the observed results, these results strongly suggest that SNORDs can guide 2′-*O*-methylation of mRNA to regulate gene expression, with consequences for protein expression and physiologic enzyme activity. Taken together with the results from *Fbl* knockdown in HeLa cells and the snoRNA-specific findings in our genetically engineered 293T cells, results from this mouse model suggest that *Pxdn* mRNA is targeted for Nm modification by at least *U32A* and *U51*.

### SnoRNA-guided modification of *Pxdn* mRNA inhibits translation

One explanation for the discordance between the reduced mRNA levels and increased protein expression is that snoRNA-guided Nm on *Pxdn* mRNA inhibits translation, and loss of the modification significantly increases translational efficiency. Prior work using synthetically-modified RNA templates has shown that Nm modification of a single codon has a moderate to strong inhibitory effect on translation, particularly at the second position of a codon^7–9^.

**Fig. 4:**
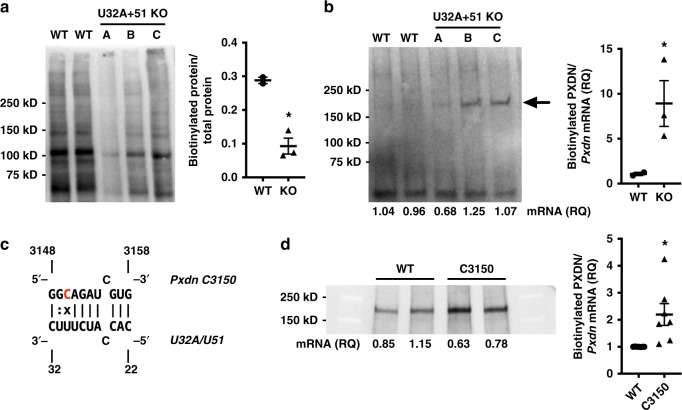
Nm modification of mRNA codon inhibits translation. **a**, **b** 293T WT and *U32A* + *U51* KO cells transfected with myc-tagged *Pxdn* and labeled with AHA for 4 h. Plots show mean ± SEM. **a** AHA-labeled protein detected by biotin click-labeling. Plot shows quantification, normalized to total protein (*p = 0.0083). **b** PXDN protein captured by anti-myc IP, then click-labeled with biotin and detected by immunoblotting (arrow). Plot shows quantification of biotinylated PXDN normalized to relative mRNA expression and total biotinylated protein from Fig. 4a (*p = 0.0485). **c** Mutation of *Pxdn* A3150 to C3150 (red) creates a base-pair mismatch at the putative methylation site. **d** 293T WT cells were transfected with myc-tagged *Pxdn* WT or C3150 mutant. Cells were AHA labeled for 4 h, then nascently translated PXDN protein was captured, biotinylated, and quantified as in **b**. *n* = 7/group, across three independent experiments. Plot shows mean ± SEM, *p = 0.0119 vs. WT by unpaired *t*-test. Representative immunoblot shown, including relative *Pxdn* mRNA expression levels (RQ)

To test this effect on a full-length mRNA in living cells, we performed metabolic pulse-labeling of protein synthesis in our 293T cells, using the non-radioactive amino acid analog, l-aziodohomoalanine (AHA)^28^. Incorporated AHA is then biotinylated using click-chemistry, allowing for detection and quantification of nascent proteins. Compared to the parental (WT) 293T cell line, *U32A* + *U51* KO cells showed lower rates of total protein synthesis, as measured by AHA incorporation and biotinylation (Fig. 4a). This is consistent with our observation that the snoRNA KO cells proliferate more slowly than WT, and may be due to a loss of *U32A*- and *U51*-guided Nm modification on 28S rRNA. To specifically assess the rate of *Pxdn* mRNA translation in these cells, we overexpressed myc-tagged human *Pxdn*, and again pulse-labeled with AHA. PXDN protein was then captured by immunoprecipitation and metabolically labeled protein was biotinylated. As shown in Fig. 4b, the amount of nascent PXDN is greater in *U32A* + *U51* cells than WT (arrow). When normalized to total biotinylated protein production and *Pxdn* mRNA expression, our results show that significantly more nascent PXDN protein is produced per mRNA transcript in *U32A* + *51* snoKO cells than WT (Fig. 4b, graph). These data support the hypothesis that snoRNA-guided Nm modifications on mRNA inhibit translation in living cells.

Next, we mutated the putative *U32A*/*U51* target site on *Pxdn* mRNA to test whether Nm modification at A3150 mediates translational inhibition. All known box C/D snoRNAs have canonical Watson-Crick base pairing with their target at the methylation site^29^, and mutation of the target site to create a base-pair mismatch has been shown to disrupt target methylation^30^. We therefore mutated the putative *Pxdn* target site from A3150 to C3150 to create a C:U mismatch with *U32A* and *U51* and disrupt snoRNA-guided methylation (Fig. 4c). *Pxdn* WT and C3150 constructs were then transfected into 293T WT cells, and *Pxdn* mRNA translation was assayed with metabolic labeling as above. The results in Fig. 4d show that the *Pxdn* C3150 has higher levels of translation. This is consistent with the A3150 site being a target of snoRNA-guided Nm modification, as a single nucleotide mutation at this position promotes translation.

### Molecular modeling of Nm modification in ribosomal A-site

**Fig. 5:**
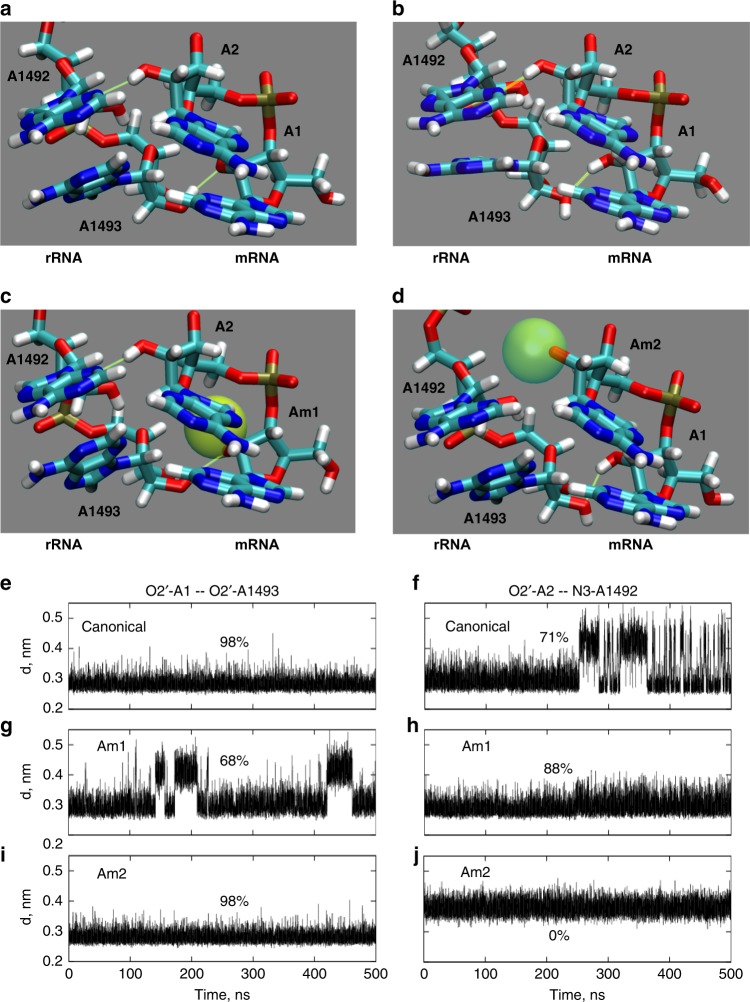
Molecular dynamics modeling of *Pxdn* Nm codon modification at the ribosomal A-site. SnoRNA-guided Nm modification on *Pxdn* mRNA is predicted at the first position of an AAG lysine codon. Our model of the ribosomal A-site included the mRNA, tRNA, and rRNA, but only the first two nucleotides of the AAG codon (A1 and A2) and the relevant monitoring rRNA nucleotides (A1492 and A1493) are shown for clarity. Hydrogen bonds are shown as yellow lines. Movies of the full trajectories are available online. **a**, **b** Representative configurations of the canonical interaction. For the A1:A1493 interaction, note that A1 can serve as the hydrogen bond donor (**a**) or acceptor (**b**). **c**, **d** Representative configurations of the Am1 and Am2 modifications, respectively, where the van der Waals radius for the Nm methyl group is shown as a yellow-green sphere. **e–j** The A1:A1493 and A2:A1492 donor-acceptor distances are shown for 500 ns simulations. Distances < 0.32 nm are consistent with hydrogen bonding interactions, and the percent of time that each interaction falls within this range are also shown. **e**, **f** canonical (unmodified). **g**, **h** Am1 (Am at first codon position). **i**, **j** Am2 (Am at second codon position)

Two groups have shown that codon context influences the magnitude of Nm-induced translational stalling^7–9^. Building on that work, we performed molecular dynamics simulations of a lysine codon in the A-site of a ribosome. Without Nm modification at the codon, the canonical interaction between mRNA backbone and rRNA monitoring bases can form two interchangeable conformations (Fig. 5a, b and Supplementary Movie 1). That is, the hydrogen bond between the A1:A1493 2′-OH groups is dynamic, with either group being able to serve donor or acceptor. Despite this, there is a relatively fixed interaction distance, consistent with stable hydrogen bonding (<0.32 nm for 98% of the simulation, Fig. 5e). In contrast, the hydrogen bond of A2:A1492 is less stable (Fig. 5f). When A1 is methylated to Am, as predicted in the case of *Pxdn*, the 2′-*O*-methyl now sterically restricts the Am1:A1493 interaction such that A1493 must be the electron donor, and the Am1:A1493 donor-acceptor distance is destabilized (Fig. 5c, g and Supplementary Movie 2). At the same time, the adjacent A2:1492 hydrogen bond is somewhat stabilized relative to the unmodified configuration (compare Fig. 5f, h)—though our biological data suggest this is insufficient to compensate for the disruption at Am1:A1493. We also modeled the effect of Nm at the second position of the same codon, which is thought to be strongly inhibitory to translation^7–9^. In this case, Nm at the 2nd position has no effect on the A1:A1493 interaction (Fig. 5i) but causes a steric hindrance that breaks the A2:A1492 hydrogen bond altogether (Fig. 5d, j, and Supplementary Movie 3). Overall, these simulations predict an additional level of detail that is not evident in the recent crystal structure^7^. Based on these results, we hypothesize that Nm modifications could disrupt a wide range of other RNA interactions that are dependent on 2′-OH groups.

## Discussion

**Fig. 6:**
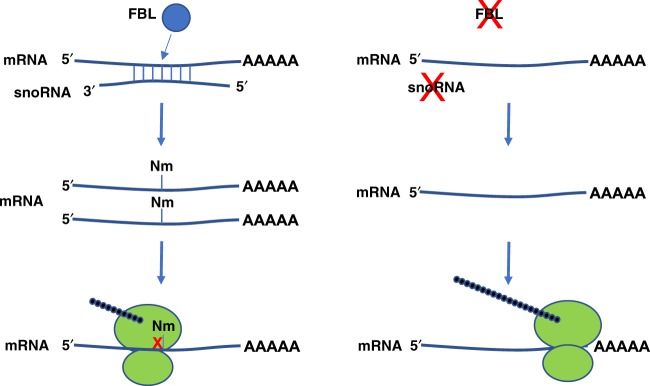
Proposed model. Our findings suggest that *Pxdn* mRNA is targeted by snoRNAs for FBL-mediated Nm modification. The presence of this Nm modification in the coding sequence leads to an increase in mRNA abundance but with suppressed translation (left). In the absence of either FBL or the snoRNA guide(s), the mRNA is not Nm-modified, resulting in lower mRNA expression, but the translational suppression is released, leading to paradoxically higher levels of protein expression (right)

Collectively, our results show that Nm modifications can occur on mRNA beyond the 5′-cap, and that at least some of these modifications are catalyzed by fibrillarin and guided by box C/D snoRNAs. Whether or not Nm modifications are widespread throughout the mRNA transcriptome, our data suggest that Nm can both stabilize mRNA and inhibit its translation in both cultured cells and animals (Summarized in Fig. 6). These findings reported here therefore expand our knowledge of epitranscriptomic modifications by providing in vitro and in vivo validation of ribose modification on mRNA as a novel mechanism of genetic regulatory control. Finally, our work also raises new questions for future study. For example, it is unclear where and when snoRNA-guided Nm modification happens during the mRNA life-cycle. Dai et al. reported some intronic Nm sites, suggesting that the modification of pre-mRNA can occur in the nucleus^6^. Additionally, prior work has demonstrated that snoRNAs can translocate to the cytoplasm during cellular stress, raising the possibility that Nm modifications could be added dynamically on mature mRNA, if sufficient amounts of the FBL complex proteins are also present in the cytoplasm^20^.

## Methods

### RNA and RT-qPCR

Total RNA was isolated using TRIZOL, per manufacturer’s directions. All PCR analyses were performed and analyzed with technical duplicates, using PowerSybr (ThermoFisher) and a StepOnePlus (ABI) Real-Time PCR System.

### UPLC MS/MS

Nucleosides were generated from 100 ng total, poly-A selected (Promega), and rRNA-depleted (NEB) as previously described^31^. Briefly, digestion was performed with nuclease P1 (Sigma, 2U) in buffer containing 25 mM NaCl and 2.5 mM ZnCl2 for 2 h at 37 °C, followed by incubation with Antarctic Phosphatase (NEB, 5U) for an additional 2 h at 37 °C. Nucleosides were separated and quantified using UPLC-MS/MS as previously described, except acetic acid was used in place of formic acid^32^.

### Cell culture

HeLa (ATCC CCL-2), 293T (ATCC CRL­3216). and 293T CRISPR KO clones were maintained in MEM (ATCC) or DMEM (ATCC) supplemented with 10% FBS (Corning) from passages 120–130 or 32–55 respectively. FBL was knocked down using Silencer Select siRNA (ThermoFisher s4822) at a concentration of 5 µM for 48 h using Lipofectamine RNAiMAX and OptiMEM (ThermoFisher) according to manufacturer instructions. VPO1 overexpression construct was transfected using Lipofectamine 2000 for 24 h according to manufacturer instructions. RNA and protein were extracted from fresh cells as described above.

### Reverse transcription at low dNTP followed by PCR

This procedure was adapted from the previously described RTL-P method^25^. cDNA was generated from 500 ng of total RNA, with oligo-dT RT priming, under two conditions: (1) standard SuperScript III (ThermoFisher) reverse transcriptase reaction (1 mM dNTP mix), and (2) under the same conditions except low dNTP concentration (0.1 mM dNTP mix). qPCR was then performed in 20 μL reactions with Power SYBR, 0.25 µM forward and reverse primers (both 5′ to the putative Nm site, as shown in Fig. 1d and detailed in Supplementary Table 1), and 2 μL of each cDNA with a hot start (95 C; 10′) and 40 cycles of 95 C (15 s) and 60 C (1′). Relative quantification of *Pxdn* from each cDNA reaction (both high and low dNTP) was first normalized to an internal reference gene (*Rplp0*) to correct for any differences in total RNA input (standard ddCT method). The ratio of low/high dNTP product was then calculated for each condition, to normalize the low dNTP result to the amount of *Pxdn* mRNA in each sample. Finally, this value could be compared between experimental (KO or FBL KD) conditions as a relative quantity (RQ). This method accounts for changes in transcript methylation relative to transcript abundance in normal conditions.

### Digital PCR

QuantStudio 3D Chips and the ProFlex PCR System (ThermoFisher) were used to determine gene copy number. cDNA was made using 0.5–3 μg of RNA with restricted or standard dNTPs as described above. Reactions were made with QuantStudio 3D Digital PCR Master Mix (ThermoFisher), 5 μL of cDNA, 250 nM RT PCR primers, and supplemented with 1X SYBR Green 1 dye (ThermoFisher). Digital PCR was performed with a hot start (96 C; 10′), 39 cycles of 60 C (2′) and 98 C (30 s), and 1 cycle of 60 C (2′). Chips were read with the QuantStudio 3D Digital PCR Instrument (ThermoFisher) and analyzed using QuantStudio 3D Analysis Suite Cloud Software (ThermoFisher).

### Primer sequences

Primers are shown in Supplementary Table 1.

### SnoRNA knockout cells

*SNORD32A*- and *SNORD51*-targeting sgRNAs were designed using CHOPCHOP^33^ and Cas-OFFinder^34^ and cloned into PX458 (Addgene #48138)^35^. Cultures of 293T cells were first transfected with pairs of sgRNAs engineered to introduce a 44 base deletion in *SNORD32A* or a 46 base deletion in *SNORD51* using TransIT-LT1 (Mirus), according to manufacturer’s instructions. Twenty-four hours post transfection, GFP-positive cells were sorted using a Beckman-Coulter Astrios and single cells were deposited into wells of a 96-well plate. Individual clones were expanded and screened by PCR for the desired deletion event. Clones that were positive by PCR screening were then verified by cloning PCR products into pCR4-TOPO (ThermoFisher) and Sanger sequenced. The 293T *SNORD32A* knockout clone #8 was then used to create the double knockout by introducing the pair of *SNORD51* sgRNAs. Single cell clones of the resulting *U32A* + *U51* double knockouts were isolated and screened by PCR, followed by sequencing to verify deletions.

### Site-directed mutagenesis

To generate C3150 construct, *Pxdn* WT plasmid was mutated using the PCR-based Q5-Site-Directed Mutagenesis Kit (New England BioLabs) according to manufacturer’s instructions. The primer set used to introduce the mutation (included in Supplementary Table 1) was designed with the online NEBaseChanger software provided by New England BioLabs. DNA sequencing of the construct confirmed the single nucleotide mutation.

### Peroxidase activity

Peroxidase activity was measured using the Amplex® Red Hydrogen Peroxide/Peroxidase Assay Kit (ThermoFisher A22188). In brief, cell and cardiac lysates were prepared in cold phosphate-buffered saline containing 1% hexadecyltrimethylammonium bromide (Sigma, H9151) with cOmplete™ Protease Inhibitor Cocktail (Roche 4693116001) and homogenized with a glass dounce tissue grinder. Samples were prepared according to manufacturer’s instructions. Peroxidase activity was determined with technical duplicates using a TECAN SpectraFluor Plus plate reader and normalized to sample protein concentration.

### Animals

Mice were maintained under standard husbandry conditions and IACUC regulations and in compliance with all ethical regulations, as approved by Duke University. Use of these animals received ethical approval from IACUC at Duke University. The *RPL13a* snoRNA KO mice were generated by the Schaffer Laboratory at Washington University in St. Louis^19^, and obtained for use under a Materials Transfer Agreement. Upon sacrifice, mouse hearts were flash frozen. Hearts were then homogenized for RNA and protein preparations.

### Western blot

Cell and mouse heart lysates were prepared in RIPA buffer (Sigma R0278) with cOmplete™ Protease Inhibitor Cocktail (Roche 4693116001). Protein concentrations were determined using a BCA assay (ThermoFisher 23225). Proteins were separated on 4–15% SDS-PAGE gels and transferred to nitrocellulose membranes. Immunoblotting was performed with the following antibodies: anti-fibrillarin (Abcam ab166630, 1:5000), and anti-Pxdn (Millipore ABS1675, 1:500). Images were captured using a ChemDoc XRS + system (Bio-Rad). Protein expression levels were normalized to total protein imaging and analyzed using ImageLab software (Bio-Rad).

### Azidohomoalanine labeling

*Pxdn* overexpressed 293T cells were incubated with 50 µM AHA (ThermoFisher C10102/ Click Chemistry Tools 1066) in methionine-free DMEM for the indicated time followed by lysate preparation and protein quantification as previously described. To quantify nascent PXDN protein, PXDN was pulled down with a myc-tagged antibody (CST 2276S). Both PXDN and total protein lysate then were click-labeled with biotin (ThermoFisher B10185) using the Protein Reaction Buffer Kit (ThermoFisher C10276/ Click Chemistry Tools 1262) according to the manufacturer’s instructions. Biotin-labeled samples were analyzed by western blot using Anti-Biotin-HRP (CST 7075S, 1:1000). The total nascent protein from cell lysate was normalized to total protein level as loading control; nascent PXDN level following pull-down was normalized to total nascent protein level and mRNA transcript levels.

### Statistical analysis

The sample size chosen for our animal experiments in this study was estimated based on our prior experience of performing similar sets of experiments. No statistical methods were used to predetermine sample size. All animal results were included and no method of randomization was applied. The experimenters were not blinded to allocation during experiments and outcome assessment. Cell culture experiments were independently performed at least three times. For all the graphical plots, data are expressed as mean ± SEM. Statistical analyses were performed using GraphPad Prism 8. Differences were analyzed by unpaired *t*-test or one-way ANOVA with Dunnett’s correction for multiple comparisons, as indicated. *p*-values ≤ 0.05 were considered significant. The sample sizes (biological replicates), specific statistical tests used, and the main effects of our statistical analyses for each experiment are detailed in each figure legend.

### Molecular dynamics simulations

We performed molecular dynamics (MD) simulations to understand the effect of 2′-*O*-methyl modification on the interaction of the codon nucleosides at the A-site of the ribosome. We obtained the initial structure from the PDB databank (PDB ID: 1XMQ) of the anti-codon stem-loop of tRNA-Lys3 bound to the AAA codon at the A-site of the ribosome^36^. For the purposes of our simulations, we included the mRNA, tRNA ASL and the sections of ribosomal RNA that are neighboring the mRNA–tRNA complex (150 nucleotides) along with coordinated Mg^2+^ ions. We used the AMBER99 force-field^37^ for the RNA with Chen-Garcia corrections for the nucleobases^38^ and Bergonzo-Cheatham corrections for the Van der Waals radii of the backbone oxygens^39^. TIP4P model with Ewald-optimized ion parameters was used for the water^40^. AMBER-type force-field parameters for the atoms of the modified nucleoside were generated. For obtaining the partial charges on the atoms, we used the online RESP charge-fitting server, RED^41^. The geometry of the modified nucleoside was energy minimized, and Hartree-Fock level theory and 6-31G* basis-sets were employed to arrive at a set of partial charges^42^. We performed three sets of simulations: (i) Canonical (ii) Am1 (Nm-modified A1), and (iii) Am2 (Nm-modified A2).

All simulations were performed using GROMACS-2016^43^. The simulation system consisted of the RNA assembly in a 1 M KCL solution in a 3D periodic cubic box. The box size was 12 × 12 × 12 nm^3^, containing ~1000 K^+^ and CL^−^ ions, and ~50,000 water molecules. The system was subjected to energy minimization to prevent any overlap of atoms, followed by a 10 ns equilibration run with the RNA heavy atoms position restrained. Following that, a 500 ns production run were performed. The MD simulations incorporated leap-frog algorithm with a 2 fs timestep to integrate the equations of motion. The system was maintained at 300 K and 1 bar, using the velocity rescaling thermostat^44^ and Parrinello-Rahman barostat^45^, respectively. The long-ranged electrostatic interactions were calculated using particle mesh Ewald (PME)^46^ algorithm with a real space cut-off of 1.2 nm. LJ interactions were also truncated at 1.2 nm. LINCS^47^ algorithm was used to constrain the motion of hydrogen atoms bonded to heavy atoms. Co-ordinates of RNA nucleotides were stored every 2 ps for further analysis. Visual Molecular Dynamics (VMD)^48^ was used to analyze the data (calculate bond-distance), visualize the trajectories, render images and movies.

### Reporting summary

Further information on research design is available in the Nature Research Reporting Summary linked to this article.

## Supplementary information

Supplementary Information

Peer Review

Reporting Summary

Description of Additional Supplementary Files

Supplementary Movie 1

Supplementary Movie 2

Supplementary Movie 3

Source Data

## Data Availability

The data that support the findings of this study are available from the authors on reasonable request. The data underlying Figs. 1–4 and Supplementary Figs. 1 and 2 are provided as a Source Data File.
