# Supplementary material for: Modification of messenger RNA by 2′-O-methylation regulates gene expression in vivo
Source: Nat Commun. 2019 Jul 30;10:3401. doi: 10.1038/s41467-019-11375-7 (PMC6667457; doi:10.1038/s41467-019-11375-7)
Supplement: Supplementary file 1 — Supplementary Information [file 41467_2019_11375_MOESM1_ESM.pdf]

Supplementary Figure 1 | Predicted *U32A*/*U51-Pxdn* interactions and validation of *Fbl* knockdown. **a**, Predicted interactions between human, mouse, and *Drosophila Pxdn* mRNA (*hPxdn*, *mPxdn*, *dPxdn*) and their respective *U32A* snoRNAs (*hU32A*, *mU32A*, *dU32A*). Nucleotide positions are numbered. *Nm modification site (Am), K = lysine codon. **b**, Peptide (RKIV) and codon sequence conservation at the predicted snoRNA-guided Nm site of *Pxdn*, in human, mouse, and *Drosophila*. **c,** Nucleotide sequence conservation of the *U32A* and *U51* snoRNA D-box antisense elements across human (h), mouse (m), and *Drosophila* (d). **d, e** Confirmation of *fibrillarin* mRNA knockdown (*FBL*) in HeLa cells, vs. negative control #1 (ctrl), using siRNA for 48h. n=3 independent experiments, SEM error bars, * $p<0.05$ by unpaired t-test. **d**, *FBL* mRNA quantification by qPCR, normalized as relative quantity (RQ) (*$p=0.0008$). **e**, FBL protein expression and quantification by immunoblotting, with quantitative normalization to total protein per lane (*$p<0.0001$). Representative immunoblot is shown.

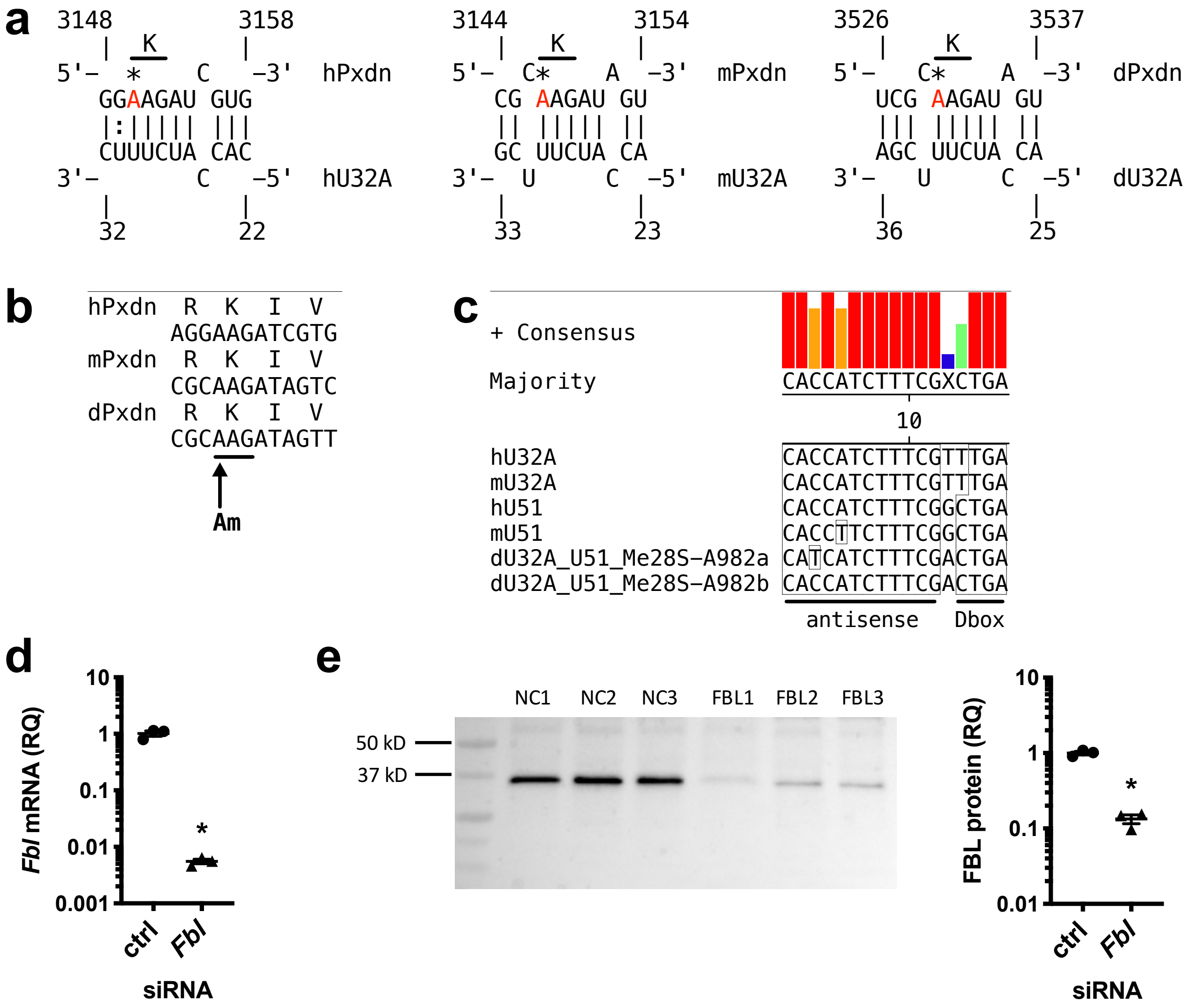

Supplementary Figure 2 | Assessment of 293T snoRNA KO cell lines and additional controls. a, CRISPR/*Cas9* methods were used in 293T cells to knockout snoRNAs *U32A* (n=3), *U51* (n=3), and *U32A+U51* (n=4). RT-qPCR demonstrated specific loss of the targeted snoRNAs, compared to parental (WT, n=2) controls. Mean and SEM error bars shown, * p<0.05 vs. WT. **b-d** *Pxdn* and two unrelated transcripts (*Gapdh* and *Pkm*) were compared in WT and *U32A+U51* KO cells. **b**, RTL-P using qPCR, with *Rplp0* as the reference transcript. *p=0.035. **c**, RTL-P using droplet digital PCR (ddPCR), which is reference-free. *p=0.007. **d**, qPCR assessment of mRNA expression levels. *p=0.014.

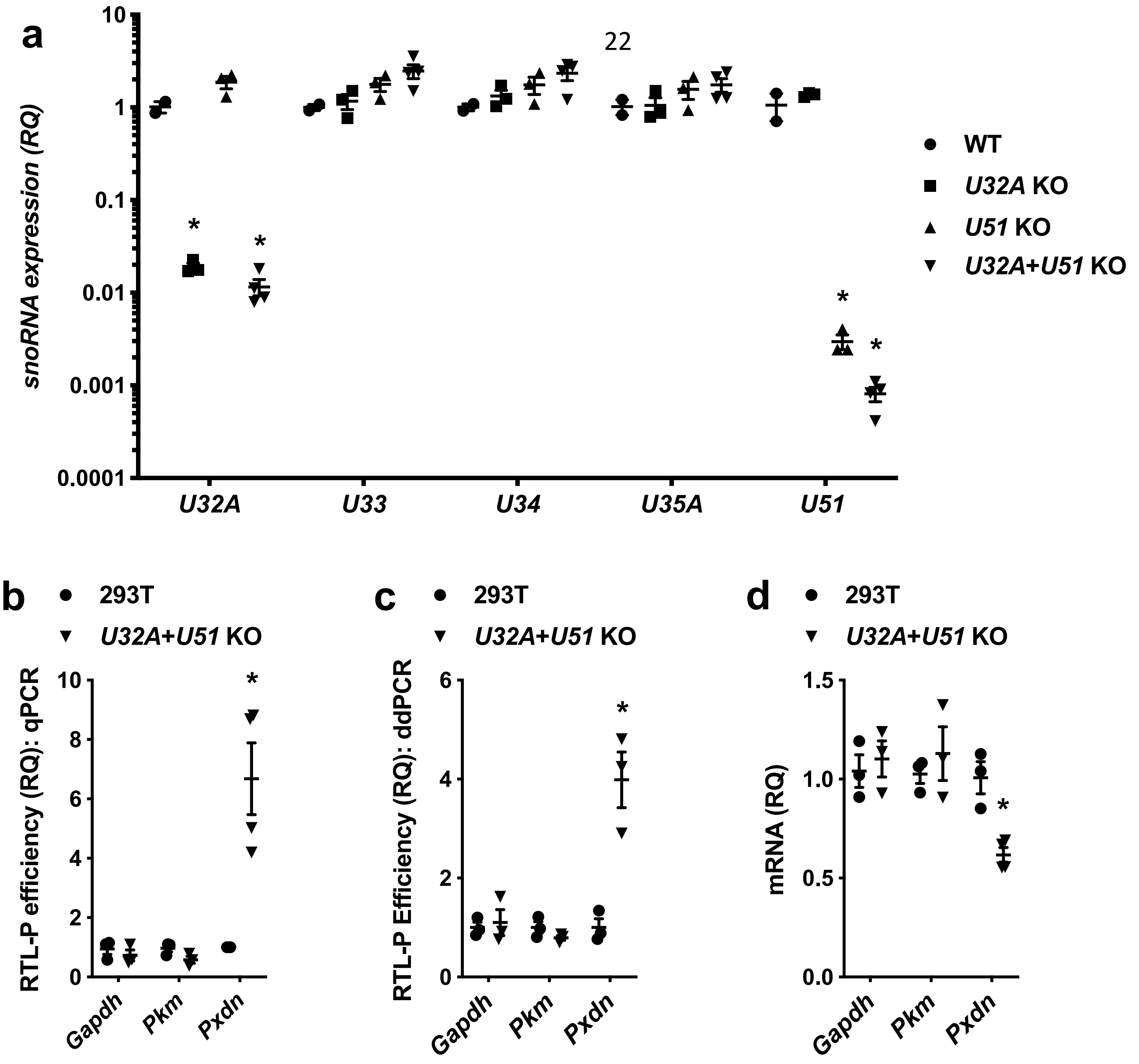

Supplementary Data Table 1: Primers and oligonucleotides (shown 5' to 3')

**Name**	**Sequence**
**mRNA**	
hGAPDH F	CTCACAGTTGCCATGTAGAC
hGAPDH R	GTTGAGCACAGGGTACTTTAT
hFBL F	GTCTATGCAGTCGAGTTCTCC
hFBL R	TCAGCAAAGATCACATCCACC
hPKM F	ACCCTCCACCTTCCATTT
hPKM R	GTGCTTCAGCTGTTGTTTATT
hPxdn F	ATGCGGGACGAGAACGA
hPxdn R	GTGGCAATGCGGTTGTG
mPxdn F	AGAACGAGAGCCCGA TACCA
mPxdn R	CCAGTGCCGGTAGGTGATGT
hRPLP0 F	GGAGACGGATTACACCTTCCC
hRPLP0 R	CAGCCACAAAGGCAGATGG
mRPLP0 F	ATCCCTGACGCACCGCCGTGA
mRPLP0 R	TGCATCTGCTTGGAGCCCACGTT
**snoRNA**	
hU32a SLRT	GCGTGGTCCCGACCACCACAGCCGCCACGACCACGCAGGTCTC
hU32a F	GGTCAGTGATGA GCAACATTCACCATC
hU33 SLRT	GCGTGGTCCCGACCACCACAGCCGCCACGACCACGCGTGGCCTC
hU33 F	GGCCGGTGATGAGAACTTCTCC
hU34 SLRT	GCGTGGTCCCGACCACCACAGCCGCCACGACCACGCGCGTCTCA
hU34 F	CGTCCATGATGTTCCGCAACTACC

hU35a SLRT	GCGTGGTCCCGACCACCACAGCCGCCACGACCACGCCTCCTGGC
hU35a F	CTTATCTCACGATGGTCTGCGGATG
hU51 SLRT	GCGTGGTCCCGACCACCACAGCCGCCACGACCACGCAATCAGAA
hU51 F	GTTGCATGATGAATAAAATCAAATCACCATCTTTCGGCT
mU32a SLRT	GCGTGGTCCCGACCACCACAGCCGCCACGACCACGCCGAGTCTC
mU32a F	GAGTCCATGATCAGCAACACTCACC
mU33 SLRT	GCGTGGTCCCGACCACCACAGCCGCCACGACCACGCACAGCCTC
mU33 F	AGCTTGTGATGAGACATCTCCCACT
mU34 SLRT	GCGTGGTCCCGACCACCACAGCCGCCACGACCACGCAGCGTCTC
mU34 F	CGTCTGTGATGTTCTGCTATTACCTACATTGTT
mU35a SLRT	GCGTGGTCCCGACCACCACAGCCGCCACGACCACGCTCCTGGCA
mU35a F	GGCAAGTGATGTCTGTTCTCACGATG
mU51 SLRT	GCGTGGTCCCGACCACCACAGCCGCCACGACCACGCCAGATTTAATTTATCA
mU51 F	GCTGAGCTCCTGATGGATTT
Universal Reverse	TCC CGA CCA CCA CAG CC
**CRISPR**	
sgRNA	
SNORD32A-1	CATCTTTCGTTTGAGTCTCA
SNORD32A-2	CTGTCATGGGCCCCCGCTGG
SNORD51-2	TCTTTCGGCTGAGTTCGTGA
SNORD51-4	AACCAGTCAGAATTTGAAAA

PCR screening for genomic deletion	
SNORD32A F	GCTGTCCTTGTCTCTGAGTCCT
SNORD32A R	TCCTCCCAGGTCCTAACTTACA
SNORD51 F	AAGATGGTGAAACCCTGTCTGT
SNORD51 R	GAATGCCACAACTGCTACCATA
**Site-directed mutagenesis**	
C3150 F	TGAGACCAGGCAGATCGTGGG
C3150 R	TAGTAGATGGTGTCGCCG
