# Supplementary material for: Modification of messenger RNA by 2′-O-methylation regulates gene expression in vivo
Source: Nat Commun. 2019 Jul 30;10:3401. doi: 10.1038/s41467-019-11375-7 (PMC6667457; doi:10.1038/s41467-019-11375-7)
Supplement: Supplementary file 3 — Reporting Summary [file 41467_2019_11375_MOESM3_ESM.pdf]

nature research

Corresponding author(s): Christopher L. Holley, MD, PhD

Last updated by author(s): Jul 1, 2019

Reporting Summary

Nature Research wishes to improve the reproducibility of the work that we publish. This form provides structure for consistency and transparency in reporting. For further information on Nature Research policies, see Authors & Referees and the Editorial Policy Checklist.

Statistics

For all statistical analyses, confirm that the following items are present in the figure legend, table legend, main text, or Methods section.

n/a	Confirmed	
☐	☒	The exact sample size (*n*) for each experimental group/condition, given as a discrete number and unit of measurement
☐	☒	A statement on whether measurements were taken from distinct samples or whether the same sample was measured repeatedly
☐	☒	The statistical test(s) used AND whether they are one- or two-sided<br>*Only common tests should be described solely by name; describe more complex techniques in the Methods section.*
☐	☒	A description of all covariates tested
☐	☒	A description of any assumptions or corrections, such as tests of normality and adjustment for multiple comparisons
☐	☒	A full description of the statistical parameters including central tendency (e.g. means) or other basic estimates (e.g. regression coefficient) AND variation (e.g. standard deviation) or associated estimates of uncertainty (e.g. confidence intervals)
☐	☒	For null hypothesis testing, the test statistic (e.g. *F*, *t*, *r*) with confidence intervals, effect sizes, degrees of freedom and *P* value noted<br>*Give P values as exact values whenever suitable.*
☒	☐	For Bayesian analysis, information on the choice of priors and Markov chain Monte Carlo settings
☒	☐	For hierarchical and complex designs, identification of the appropriate level for tests and full reporting of outcomes
☒	☐	Estimates of effect sizes (e.g. Cohen's *d*, Pearson's *r*), indicating how they were calculated

Our web collection on statistics for biologists contains articles on many of the points above.

Software and code

Policy information about availability of computer code

Data collection	Molecular dynamics simulations were performed using GROMACS-2016 (http://www.gromacs.org/). Details of the inputs and parameters are provided in the Methods of the manuscript.
Data analysis	Molecular dynamics simulations were analyzed using Visual Molecular Dynamics v.1.9.3 (http://www.ks.uiuc.edu/Research/vmd/). Statistical analyses of biological experiments were performed using GraphPad Prism 7.

For manuscripts utilizing custom algorithms or software that are central to the research but not yet described in published literature, software must be made available to editors/reviewers. We strongly encourage code deposition in a community repository (e.g. GitHub). See the Nature Research guidelines for submitting code & software for further information.

Data

Policy information about availability of data

All manuscripts must include a data availability statement. This statement should provide the following information, where applicable:

- Accession codes, unique identifiers, or web links for publicly available datasets
- A list of figures that have associated raw data
- A description of any restrictions on data availability

The data that support the findings of this study are available from the authors on reasonable request.

Field-specific reporting

Please select the one below that is the best fit for your research. If you are not sure, read the appropriate sections before making your selection.

☒ Life sciences ☐ Behavioural & social sciences ☐ Ecological, evolutionary & environmental sciences

Life sciences study design

All studies must disclose on these points even when the disclosure is negative.

Sample size	For 293T snoRNA KO cells, sample size was determined by the number of available clonal cell lines generated. For animal experiments, sample size was not pre-determined, but instead based on the number of animals available for analysis. The resulting sample sizes correspond to scientific norms, with n=3-4 clonal cell lines per KO genotype, and n=7-12 mice per het/KO genotype. We analyzed larger numbers of WT mice (n=14-23) in order to ensure that each round of testing included equal numbers of WT and genetically modified animals.
Data exclusions	No data was excluded from analysis.
Replication	All cell-based experiments were performed at least three independent times. For 293T snoRNA KO cells, each clone was considered as an independent biological sample. For mice, each mouse was an independent biological sample.
Randomization	Animals were randomly assigned to experimental groups.
Blinding	Investigators were not blinded to cell or animal genotypes. Biological samples from WT and mutant cell lines or mouse genotypes were processed together in batched assays to avoid any unintentional bias in the handling and analysis of samples.

Reporting for specific materials, systems and methods

We require information from authors about some types of materials, experimental systems and methods used in many studies. Here, indicate whether each material, system or method listed is relevant to your study. If you are not sure if a list item applies to your research, read the appropriate section before selecting a response.

Materials & experimental systems

n/a	Involved in the study	
☐	☒	Antibodies
☐	☒	Eukaryotic cell lines
☒	☐	Palaeontology
☐	☒	Animals and other organisms
☒	☐	Human research participants
☒	☐	Clinical data

Methods

n/a	Involved in the study	
☒	☐	ChIP-seq
☒	☐	Flow cytometry
☒	☐	MRI-based neuroimaging

Antibodies

Antibodies used

anti-fibrillarin (Abcam ab166630); anti-myc (CST 2276S); anti-Biotin-HRP (CST 7075S); anti-Pxdn (Millipore ABS1675)

Validation

anti-fibrillarin (Abcam ab166630), validation performed by Abcam:
"Immunohistochemistry Analysis: A 1:400 dilution from a representative lot detected VPO1 in mouse lung tissue sections (Courtesy of Guangjie Cheng, M.D., Emory University, GA, U.S.A.).
Immunoprecipitation Analysis: A representative lot immunoprecipitated all the radiolabelled VPO1 from the culture supernatant, but not from cell lysates, 20 hrs after an initial 20-min [32S]-Met pulse labeling of HEK293 cells stably expressing transfected VPO1 (Cheng, G., et al. (2011). Free Radic. Biol. Med. 51(7):1445-1453).
Western Blotting Analysis: A representative lots detected cellular VPO1 in total cell lysate and secreted VPO1 in concentrated culture medium following histone deacetylase inhibition by NaBu (Cat. No. 567430) treatment of HEK293 cells stably expressing exogenously transfected VPO1 (Cheng, G., et al. (2011). Free Radic. Biol. Med. 51(7):1445-1453).
Western Blotting Analysis: A representative lot detected VPO1 in human, mouse, and bovine sera samples, as well as a time-dependent cellular VPO1 upregulation in lysates from LPS- or TNF-alpha-stimulated HUVECs (Cheng, G., et al. (2011). Free Radic. Biol. Med. 51(7):1445-1453).): "Rabbit monoclonal [EPR10823(B)] to Fibrillarin. Suitable for: WB, IHC-P, ICC/IF, IP, Flow Cyt. Reacts with: Mouse, Rat, Human. Immunogen: Synthetic peptide corresponding to Human Fibrillarin."
See manufacturer's validation images at: http://www.abcam.com/fibrillarin-antibody-epr10823b-nucleolar-marker-ab166630.html.
Our own data show a single expected 35kD band when this Ab is used for immunoblotting, and clear loss of the band following siRNA knockdown of FBL.

anti-myc (9B11; CST 2276S), validation performed by CST: "Monoclonal antibody is produced by immunizing animals with a synthetic peptide corresponding to residues 410-419 of human c-Myc (EQKLISEEDL). Myc-Tag (9B11) Mouse mAb detects recombinant proteins containing the Myc epitope tag. The antibody recognizes the Myc-tag fused to either the amino or carboxy terminus of targeted proteins in transfected cells."
335 citations shown: https://www.cellsignal.com/products/primary-antibodies/myc-tag-9b11-mouse-mab/2276.
Our data shows no signal from mock-transfected cells that do not express myc-tagged protein.

anti-Biotin-HRP (CST 7075S), validation performed by CST: "Affinity purified goat anti-biotin antibody is conjugated to horseradish peroxidase. This product has been optimized to detect biotinylated protein markers."

56 citations shown: https://www.cellsignal.com/products/secondary-antibodies/anti-biotin-hrp-linked-antibody/7075.
Our data shows no signal from samples that were not biotinylated by click-chemistry.

anti-Pxdn (Millipore ABS1675), validation performed by Millipore: "Immunohistochemistry Analysis: A 1:400 dilution from a representative lot detected VPO1 in mouse lung tissue sections (Courtesy of Guangjie Cheng, M.D., Emory University, GA, U.S.A.).
Immunoprecipitation Analysis: A representative lot immunoprecipitated all the radiolabelled VPO1 from the culture supernatant, but not from cell lysates, 20 hrs after an initial 20-min [32S]-Met pulse labeling of HEK293 cells stably expressing transfected VPO1 (Cheng, G., et al. (2011). Free Radic. Biol. Med. 51(7):1445-1453).
Western Blotting Analysis: A representative lots detected cellular VPO1 in total cell lysate and secreted VPO1 in concentrated culture medium following histone deacetylase inhibition by NaBu (Cat. No. 567430) treatment of HEK293 cells stably expressing exogenously transfected VPO1 (Cheng, G., et al. (2011). Free Radic. Biol. Med. 51(7):1445-1453).
Western Blotting Analysis: A representative lot detected VPO1 in human, mouse, and bovine sera samples, as well as a time-dependent cellular VPO1 upregulation in lysates from LPS- or TNF-alpha-stimulated HUVECs (Cheng, G., et al. (2011). Free Radic. Biol. Med. 51(7):1445-1453)."
Our own data shows appearance of the expected 160kd Pxdn immunoblot band following overexpression of Pxdn in 293T cells.

Eukaryotic cell lines

Policy information about cell lines

Cell line source(s)	HeLa: Duke University Cell Culture facility (https://ccf.duhs.duke.edu/)<br>HEK 293T: Duke University Functional Genomics Shared Resource (https://sites.duke.edu/functionalgenomics/)
Authentication	Cell lines were authenticated by the institutional sources noted above.
Mycoplasma contamination	Cell lines were tested for mycoplasma contamination by the institutional sources noted above.
Commonly misidentified lines<br>(See ICLAC register)	N/A

Animals and other organisms

Policy information about studies involving animals; ARRIVE guidelines recommended for reporting animal research

Laboratory animals	The mice reported here are in C57BL/6J background. Both male and female mice were used for analysis, ages 8-12 weeks.
Wild animals	N/A
Field-collected samples	N.A
Ethics oversight	IACUC at Duke University.

Note that full information on the approval of the study protocol must also be provided in the manuscript.
