# Supplementary material for: Modification of messenger RNA by 2′-O-methylation regulates gene expression in vivo
Source: Nat Commun. 2019 Jul 30;10:3401. doi: 10.1038/s41467-019-11375-7 (PMC6667457; doi:10.1038/s41467-019-11375-7)
Supplement: Supplementary file 4 — Description of Additional Supplementary Files [file 41467_2019_11375_MOESM4_ESM.docx]

**Description of Additional Supplementary Files**

File Name: Supplementary Movie 1
Description: Molecular Dynamics Model of Codon AAG in Ribosomal A-site. Canonical rRNA-mRNA interaction at ribosomal A-site, showing the H-bonds of A1:A1493 and A2:A1492. For the A1:A1493 interaction, A1 can serve as the donor or acceptor.

File Name: Supplementary Movie 2
Description: Molecular Dynamics Model of Modified Codon AmAG in Ribosomal Asite. Nm modification of A1 (Am1), shown in yellow, does not abrogate the A1:A1493 H-bond, but now the H-bond interaction is unidirectional, with A1493 as the obligate donor and Am1 as the obligate acceptor

File Name: Supplementary Movie 3
Description: Molecular Dynamics Model of Modified Codon AAmG in Ribosomal Asite. Nm modification of A2 (Am2), highlighted in yellow, completely breaks the A2:A1492 H-bond.
