# Supplementary material for: Modification of messenger RNA by 2′-O-methylation regulates gene expression in vivo
Source: Nat Commun. 2019 Jul 30;10:3401. doi: 10.1038/s41467-019-11375-7 (PMC6667457; doi:10.1038/s41467-019-11375-7)
Supplement: Supplementary file 2 — Peer Review [file 41467_2019_11375_MOESM2_ESM.pdf]

Reviewers' comments:

Reviewer #1 (Remarks to the Author):

The manuscript “Modifications of messenger RNA by 2’-O-methylation regulates gene expression in vivo” describes the methylation of mRNAs by SNORDs as potential regulatory mechanism for protein synthesis. The authors convincingly show that the snoRNA U32A or/and U51 target and methylate the Pxdn mRNA and thereby destabilize the mRNA but, strikingly, increase the synthesis of the respective protein. To my knowledge this is the first study directly demonstrating mRNA methylation by snoRNAs and the associated protein complex. For researches in this field this is exceptionally interesting.
This study is well designed and carried out and subsequently the manuscript is clearly written. However, some points might be added to potentially improve the quality of the manuscript.

#1: Knock outs of U32A and also of U32A/U51 showed a decrease in methylation levels of the mRNA and increased expression of PDXN. Form the RTL-P experiments it seems that U51 significantly adds to the methylation level of the mRNA. To my opinion, it would be very interesting to study U51 knock out cells as well and not only the double knock out of U32A and U51.

#2: How efficient is the methylation by snoRNPs? Is the respective nucleobase quantitatively methylated? If only small amounts of mRNA carry the 2’ O methylations than the regulatory role of snoRNAs might only be a small one. The authors use RTL-P to quantify the methylation levels so maybe it is possible to estimate the methylation levels. Along this line, I do not understand the scales the authors use for depicting their results of RTL-P. For example, what does it mean if you have an “RTL-P efficiency” of 15? Is it a relative value? Maybe the authors can improve the figure legends to clarify this (in Figures 1e or 2a).

Minor Points:

#3: Since the authors showed in a previous publication that the snoRNA U32A is also exported into the cytoplasm it would be interesting to maybe comment on this. Is it possible that the mRNA is methylated in the cytoplasm or would the authors exclude this?
Where is it modified if exported to cytoplasm?

#4: In Figure 2d the methylation levels of total RNA, polyA RNA and rRNA depleted RNA are shown. What does oligo dT x2 mean. Was the purification carried out two times? Please add the information to the figure legend.

#5: Why is PXDN protein not detectable although it should be there? Is there an explanation?

#6: The authors should add a reference to the AHA-pulse labeling (line 128).

#7: I would strongly recommend changing the background color of Figure 4a-d. Green/green is unfortunate.

Reviewer #2 (Remarks to the Author):

The authors reported that internal 2’-O-methylation (Nm) of mRNA could regulate mRNA abundance and protein levels in cell lines and mouse liver, using Pxdn mRNA as an example. They showed that snoRNA U32a and U51, as well as FBL, are required for the Nm modification on Pxdn, and perturbing FBL or the snoRNA expression decreases Pxdn mRNA level and increases its protein synthesis. They then used molecular dynamics simulation to predict how Nm on codon could affect rRNA and mRNA recognition and thus affect translation.

The authors demonstrated one of the first cases of how mRNA internal Nm affects RNA level and protein production in vitro and inside cells, and an additional cellular function of snoRNA to guide mRNA Nm modification. These discoveries are novel. They proposed that snoRNA-guided mRNA Nm modification could be a widespread mechanism of post-transcriptional gene regulation, which is of interest to the growing community of epitranscriptomics. We suggest a few additional controls and experiments to strengthen the authors' claims.

1. The idea of snoRNA-guided and FBL-mediated mRNA internal Nm methylation should be further confirmed. The only evidence of interaction between snoRNA U32A and Pxdn mRNA came from the published work (ref. 22 of the manuscript), where they identified a few chimeric reads supporting the interaction. snoRNA U51 were proposed by the authors to have similar function as U32A based on their sequence similarity, while the interaction between U51 and Pxdn remains to be determined. An assay of Pxdn mRNA pull down and RT-qPCR of the two snoRNA species (with control snoRNAs that should not bind) might be needed to validate the RNA-RNA interaction.

2. Related to Figure 1d, the authors simplified the RTL-P method. I would be careful about this assay since the changes of Nm level on Pxdn mRNA in this manuscript was all based on this assay. (i) the RT primers used in this assay was not specified in either Fig 1d nor the method section. (ii) It is described in the method section of RTL-P that "Gene reverse transcriptase efficiency of cDNA made under dNTP restricted conditions was calculated as a ratio to cDNA made under standard conditions and normalized to a reference gene (RPLP0)." The key of RTL-P is to quantify the RT-stop caused by Nm under restricted dNTP conditions. However, different from the original setup in ref 23, the authors instead calculated the RT efficiency as the ratio of cDNA products between low and high dNTP concentrations. Please justify. (iii) The authors should include negative control genes where siFBL or siU32A/51 does not affect "RTL-P efficiency". (iv) A 15-fold change in RTL-P efficiency after siFBL (Fig. 1e) suggests that the proposed 3150Am site is almost fully Nm-methylated (1-1/15 = ~95%). If this is the case, maybe the authors could even directly visualize the RT-stop product.

3. It is really interesting to see in Figure 3 that while U32A+U51 KO cells have lower overall protein synthesis, the production of myc-tagged hPXDN is increased. Based on this, the author concluded that the Nm modification inhibit mRNA translation. More experiments are needed to support this conclusion: (i) The amount of myc-tagged hPXDN was normalized to the amount of biotin-labelled protein, while the potential difference in the amount of hPXDN mRNA (for example, caused by the difference in transfection efficiency) was not considered. (ii) To claim that it is the U32A/U51-mediated Nm modification that affects translation, myc-tagged hPXDN constructs with mutations at the proposed 3150Am site (or around it) may need to be tested in the same assay to see if the translation upregulation is abolished or not.

Minor points:
1. In the abstract, N6-methyladenosine (m6A) was exemplified as a post-transcriptional gene regulator for translation. Wang et al's work on YTHDF1, one of the first mechanistic studies on m6A's function on translation, should be cited as well.
2. In Figure 1b, the calculation of each of the Nm level, i.e., the y axis of the figure, should be specified. A "mock" sample with only digestion enzymes but no experimental RNA should be included to account for potential Nm nucleosides from the reagents.

Reviewer #3 (Remarks to the Author):

In this manuscript, the authors use a combination of experimental and computational techniques to explore the physiological role of m6A modifications in mRNA. With regards to the computational

aspects of the manuscript, the authors perform multiple 50ns explicit-solvent simulations of the decoding center of the ribosome (tRNA ASL, mRNA and surrounding ribosomal atoms). Since the current calculations were not probing large-scale conformational dynamics, these simulations of a portion of the ribosome are appropriate to explore the local influence of mRNA modifications. The forcefields and quantum mechanical techniques applied are consistent with best practices in the field. Based on these simulations, the authors argue that the unmodified RNA has more conformational variability, which may help stabilize the system through an increase in entropy. When the modification is introduced, the authors argue that the modification sterically impedes the mRNA-tRNA interactions. While the claims are reasonable, there are some issues that should be addressed.

1) It does not appear that there is any quantitative analysis of the simulations. Rather, there are only a few snapshots and movies shown, along with some qualitative commentary based on visual inspection of the simulations. While the claims may very well be valid, there is no reason for such a qualitative presentation of the results. Further, it is not clear why the simulations were only performed for 50ns, which is much less than current standards in the field. As the authors are certainly aware, it is no uncommon for systems to require hundreds of nanosecond to properly equilibrate. With modern resources, such a simulation should probably only take about a week on a single compute node (with a GPU). With the relatively low cost of these simulations, it would be worthwhile to significantly extend the duration of the simulations, and perform of systematic analysis of the dynamics (e.g. interatomic distance distributions would be helpful).

2) The caption to figure 4 needs to be substantially improved. As written, there is a lack of scientific content in the caption. Here are a couple examples of how to improve this caption.
- It is not stated how these particular snapshots were chosen. Are they representative, or are they the initial configurations after energy minimization?
- There is a statement "Full simulations available as online movies..." should read "Movies of the full trajectories are available..."
- There is no description provided for the arrows and large green spheres.
- Is panel e a schematic based entirely on the simulations, or is this a synthesis of all results in the study? If it is a synthesis of the full study, it should be included as a separate figure.
- A full description of panel e should be provided. There are six subpanels, which deserve a full explanation.

NCOMMS-18-16019-T
Point-by-point response to Reviewers

Reviewer #1:
#1: Knock outs of U32A and also of U32A/U51 showed a decrease in methylation levels of the mRNA and increased expression of PXDN. From the RTL-P experiments it seems that U51 significantly adds to the methylation level of the mRNA. To my opinion, it would be very interesting to study U51 knock out cells as well and not only the double knock out of U32A and U51.

This is an excellent suggestion, as it is possible that *U51* KO cells might reveal that *U51* is the "dominant" snoRNA in the phenotype of *U32A+U51* double-knockout cells.

We therefore generated three independent *U51* KO cell clones and analyzed them for *Pxdn* mRNA methylation, mRNA quantity, and peroxidase activity (as we did previously with *U32A* and *U32A+U51* KO clones). Elimination of *U51* alone had no significant effect on *PXDN* methylation, mRNA expression, or enzyme activity (see updated **Fig. 2a-c** and highlighted text in lines 78-89). Therefore, isolated loss of either *U32A* **or *U51*** has little or no effect on *Pxdn* expression, while the loss of both *U32A* and *U51* results in strong changes to *Pxdn* mRNA levels, Nm modification, and enzyme activity. Since *U32A* and *U51* share nearly identical antisense elements for guiding 2'-*O*-methylation (Extended Data Fig. 1c), but no other sequence elements, these new results further support our hypothesis that *U32A* and *U51* redundantly guide Nm modification of *Pxdn* mRNA.

#2a: How efficient is the methylation by snoRNPs? Is the respective nucleobase quantitatively methylated? If only small amounts of mRNA carry the 2’ O methylations than the regulatory role of snoRNAs might only be a small one. The authors use RTL-P to quantify the methylation levels so maybe it is possible to estimate the methylation levels.

The stoichiometry of modifications on mRNA is an important and difficult question. Our genetic models strongly suggest that the regulatory role of the snoRNAs is substantial, even though we cannot measure the exact amount of modification.

Nm modifications of individual RNAs have classically been detected by primer extension, but the RT inhibition at Nm sites is not 100% (see primer extension results below). As such, the method cannot directly measure the stoichiometry of modifications, but can be only used to determine <u>relative</u> amounts of modification (when normalized to the amount of target RNA in each sample). RTL-P is simply a variation of the primer extension method that allows for PCR amplification of the RT products, which vastly increases the sensitivity for detecting Nm sites on low-abundance targets.

An alternative approach, the method of RiboMeth-seq, can provide somewhat accurate estimates of Nm stoichiometry, but only for abundant targets in combination with very deep RNA sequencing (750-1000x)[1,2]. We have attempted RiboMeth-seq on mRNA from HeLa cells,

but were unable to attain sufficient sequencing depth to map Nm sites on *Pxdn* mRNA, even after using two full lanes of HiSeq4000 for a single sample. In fact, despite 834M reads mapping to mRNA, the average read depth per *Pxdn* nucleoside was still <10. This result, and other results using digital PCR (ddPCR, detailed below), suggest that *Pxdn* mRNA is expressed at low levels, making it a particularly difficult target to analyze. We apologize that we have been unable to provide the quantitative assessment requested by the Reviewer.

#2b. Along this line, I do not understand the scales the authors use for depicting their results of RTL-P. For example, what does it mean if you have an "RTL-P efficiency" of 15? Is it a relative value? Maybe the authors can improve the figure legends to clarify this (in Figures 1e or 2a).

We chose the term "RTL-P efficiency" since the assay reports on the efficiency of the reverse transcription reading through Nm sites, and the Reviewer is correct that we are reporting relative values. Without Nm modifications, the RT reaction proceeds at higher efficiency, resulting in relatively more qPCR product for a given amount of RNA template. In the presence of Nm modification, the RT has relatively lower efficiency, resulting in less qPCR product. We find it helpful to report this as a normalized ratio between two conditions being studied, in order to easily visualize the relative difference in their "RTL-P efficiency" (which reflects the relative amount of Nm modification). Since we are reporting the results as a normalized ratio between the experimental and control groups, we have now changed this label to "RTL-P efficiency (RQ)" and added the following statement to the Figure Legend:

The amount of qPCR product from the low dNTP reactions of two different samples can be compared to establish the relative "RTL-P efficiency (RQ)". RQ = relative quantity. See Methods for detail.

We hope that this clarifies that the results are relative quantities between the control and experimental condition. For example, the "RTL-P efficiency (RQ)" in **Fig 1e** shows that around 15x more PCR product was generated in reactions amplifying the specified segment of *Pxdn* mRNA following FBL knockdown, compared to the control siRNA. We have also now included a more detailed description of the assay and calculation of the relative RTL-P efficiency in the manuscript Methods (see response to Reviewer #2 below).

Minor Points:
#3: Since the authors showed in a previous publication that the snoRNA U32A is also exported into the cytoplasm it would be interesting to maybe comment on this. Is it possible that the mRNA is methylated in the cytoplasm or would the authors exclude this?
Where is it modified if exported to cytoplasm?

Knowing that snoRNAs and snoRNPs can translocate to the cytoplasm under certain conditions, it is plausible that 2'-*O*-methylation could occur in the cytoplasm. As noted by the Reviewer, box C/D snoRNAs can accumulate in the cytoplasm during stress[3]. Dynamic translocation of snoRNAs into the cytoplasm could potentially lead to increased transcript methylation and a

selective inhibition of translation for those newly-modified mRNAs. This would be an exciting new mechanism and something worthy of future studies. We have added a brief discussion of this topic to the manuscript, lines 172-179:

Finally, our work also raises new questions for future study. For example, it is unclear where and when snoRNA-guided Nm modification happens during the mRNA life-cycle. Dai *et al*. reported some intronic Nm sites, suggesting that the modification of pre-mRNA can occur in the nucleus[4]. Additionally, prior work has demonstrated that snoRNAs can translocate to the cytoplasm during cellular stress, raising the possibility that Nm modifications could be added dynamically on mature mRNA, if sufficient amounts of the FBL complex proteins are also present in the cytoplasm[3].

#4: In Figure 2d the methylation levels of total RNA, polyA RNA and rRNA depleted RNA are shown. What does oligo dT x2 mean. Was the purification carried out two times? Please add the information to the figure legend.

In Figure 1b, "oligo dT x2," is intended to indicate that poly-A selection was carried out on the sample twice (using oligo-dT affinity capture). The legend in figure 2 has been updated for clarity:

(1) b, UPLC-MS/MS quantification of modified nucleosides on total RNA, mRNA after two rounds of oligo-dT selection, and mRNA further purified by rRNA depletion.

#5: Why is PXDN protein not detectable although it should be there? Is there an explanation?

We were easily able to demonstrate PXDN protein by immunoblot using heart tissue (Fig. 3), but not in 293T cells (Fig. 2). In the initial publication describing *Pxdn*, the mRNA was shown to be broadly expressed across many human tissue types, while the protein appeared to be selectively expressed in the heart and vasculature (leading the authors to call the gene *vascular peroxidase 1*, or *VPO1*)[5]. There is a more recent report showing native PXDN protein expression in HEK293 cells, but the immunoblot band is quite faint, even after the authors captured PXDN from cell lysates in an IP-western approach[6]. We were unsuccessful in detecting a clear signal for PXDN protein in 293T cells with this IP-western approach, using our commercially-available antibody. Nevertheless, the published results show that PXDN protein is expressed by HEK293 cells, and we therefore hypothesize that the enzyme assay to detect peroxidase activity is simply more sensitive than immunoblotting.

#6: The authors should add a reference to the AHA-pulse labeling (line 128).

This has been added to the text (now line 126): 27. Wang, J. *et al.* Nonradioactive quantification of autophagic protein degradation with L-azidohomoalanine labeling. *Nat. Protoc.* **12**, 279–288 (2017).

#7: I would strongly recommend changing the background color of Figure 4a-d. Green/green is unfortunate.

The background has been changed to resolve this.

Reviewer #2:
1. The idea of snoRNA-guided and FBL-mediated mRNA internal Nm methylation should be further confirmed. The only evidence of interaction between snoRNA U32A and Pxdn mRNA came from the published work (ref. 22 of the manuscript), where they identified a few chimeric reads supporting the interaction. snoRNA U51 were proposed by the authors to have similar function as U32A based on their sequence similarity, while the interaction between U51 and Pxdn remains to be determined. An assay of Pxdn mRNA pull down and RT-qPCR of the two snoRNA species (with control snoRNAs that should not bind) might be needed to validate the RNA-RNA interaction.

Proof of a physical interaction between the mRNA target *Pxdn* and *U32A* and *U51* would be useful for confirming that these snoRNAs guide Nm modification in the same manner that they canonically target rRNA. We attempted to do this by pulling down *Pxdn* mRNA with biotinylated probes and using qPCR to detect *U32A*, *U51* and a negative control snoRNA *U103b*. Although we were able to selectively capture *Pxdn*, we were unable to detect any association with snoRNAs. Factors limiting this experiment might include: (1) *Pxdn* expression is quite low (as determined by RNA-seq and ddPCR), and (2) it is likely that the binding interactions between these RNAs are relatively weak (10nt duplex) and transient.

Nevertheless, the loss of Nm on *Pxdn* is seen independently with genetic loss of snoRNAs and silencing of *Fbl* expression, reasonably confirming that each component is necessary for *Pxdn* to be methylated. Additionally, we now present a new genetic model in which the *Pxdn* A3150 site is mutated to prevent snoRNA-guided modification (as suggested by this Reviewer; see Point 3 below and **Fig. 4d** of the revised manuscript). In this model, we find that the mutated *Pxdn* mRNA has increased translation, again consistent with this site being the target of snoRNA-guided modification. From a molecular genetics perspective, the ability of this single nucleotide point mutation to effectively phenocopy *U32+U51* KO is highly consistent with a direct (canonical) interaction between *Pxdn* mRNA and the *U32A*/*U51* snoRNAs.

2. Related to Figure 1d, the authors simplified the RTL-P method. I would be careful about this assay since the changes of Nm level on Pxdn mRNA in this manuscript was all based on this assay. (i) the RT primers used in this assay was not specified in either Fig 1d nor the method section. (ii) It is described in the method section of RTL-P that "Gene reverse transcriptase efficiency of cDNA made under dNTP restricted conditions was calculated as a ratio to cDNA made under standard conditions and normalized to a reference gene (RPLP0)." The key of RTL-P is to quantify the RT-stop caused by Nm under restricted dNTP conditions. However, different from the original setup in ref 23, the authors instead calculated the RT efficiency as the ratio of cDNA products between low and high dNTP concentrations. Please justify. (iii) The authors should include negative control genes where siFBL or siU32A/51 does not affect "RTL-P efficiency". (iv) A 15-fold change in RTL-P efficiency after siFBL (Fig. 1e) suggests that the

proposed 3150Am site is almost fully Nm-methylated (1-1/15 = ~95%). If this is the case, maybe the authors could even directly visualize the RT-stop product.

(i) Oligo-dT priming was used for the RT step. Although we meant for this to be communicated by the cartoon of Fig. 1d, it is now clearly stated in the Methods (Lines 199-211):

RTL-P (Reverse Transcription at Low dNTP concentrations followed by PCR). This procedure was adapted from the previously described RTL-P method[7]. cDNA was generated from 500 ng of total RNA, with oligo-dT RT priming, under two conditions: (1) standard SuperScript III (ThermoFisher) reverse transcriptase reaction (1mM dNTP mix), and (2) under the same conditions except low dNTP concentration (0.1mM dNTP mix)....

(ii) As noted by the Reviewer, our RTL-P assay is adapted from the original reference, and we have detailed our approach more clearly in the Methods (again, Lines 199-211). In that section, we have tried to more clearly describe how we achieve relative quantification of the RTL-P reactions:

... Relative quantification of *Pxdn* from each cDNA reaction (both high and low dNTP) was first normalized to an internal reference gene (*Rplp0*) to correct for any differences in total RNA input (standard ddCT method). The ratio of low/high dNTP product was then calculated for each condition, to normalize the low dNTP result to the amount of *Pxdn* mRNA in each sample. Finally, this value could be compared between experimental (KO or FBL KD) conditions as a relative quantity (RQ). This method accounts for changes in transcript methylation relative to transcript abundance in normal conditions.

For additional explanation of why we report see our results as "RTL-P efficiency (RQ)", please see response to Reviewer #1 above, in Point 2b.

(iii) As suggested by the Reviewer, we have now analyzed two new negative control genes (*Gapdh* and *Pkm*) to demonstrate the specificity of our RTL-P and mRNA qPCR results (ExtData 2b-d). As expected, the RTL-P assay for these mRNAs is not affected by *U32A+U51* KO (even when the RTL-P assay is repeated using ddPCR to obtain reference-free results).

(iv) Although we agree that it would be excellent to map the putative *U32+U51* snoRNA-guided Nm site at *Pxdn* A3150 using primer extension, we would caution against over-interpreting the result of *Fbl* knockdown with respect to this single site. The change in *Pxdn* RTL-P efficiency following *Fbl* knockdown in HeLa cells is quite a bit stronger the result from knockout of *U32A+U51* in 293T cells. Although this could simply be due to differences in cell types, we speculate that there may be other snoRNA-guided sites beyond the putative A3150 site, which are also be *Fbl*-mediated but involve other snoRNAs. In particular, the other *Rpl13a* snoRNAs may also be targeting *Pxdn.* This would be consistent with results from our *Rpl13a* snoRNA KO mice, where we see a change in Pxdn Nm content in the context of U32A KO but not U51 KO. As such, it is not at all clear what the stoichiometry of Nm modification at A3150 might be. Nevertheless, we would very much like to be the first to demonstrate the presence of Nm on an endogenously expressed mRNA using primer extension, and we have now attempted this quite

extensively. We have previously used primer extension to easily map snoRNA-guided Nm sites on (highly abundant) rRNA, including those sites targeted by the *Rpl13a* snoRNAs. However, in our many primer extension experiments to detect Nm on *Pxdn* mRNA, we have never seen a primer extension product of any kind (with zero background), presumably due to the low expression of the transcript (and potentially, low stoichiometry of Nm modification at A3150).

We therefore designed experiments to: (1) ensure our assay targeting *Pxdn* is functional, (2) determine the limit of detection for the primer extension assay, and (3) compare the results of these tests with our quantitative analysis of *Pxdn* mRNA transcript numbers (determined by ddPCR). First, we synthesized *Pxdn* RNA 100-mers, including the A3150 site and sufficient downstream sequence to perform the primer extension assay (*Pxdn* 3141-3240; see **Panel A**). For this approach, we made two 100-mers, differing only in modification at A3150: one 100% Nm methylated, and the other unmethylated. With these controls, we were able to easily detect an Nm-dependent primer extension "stop" product of 90 nt (**Panel A**).

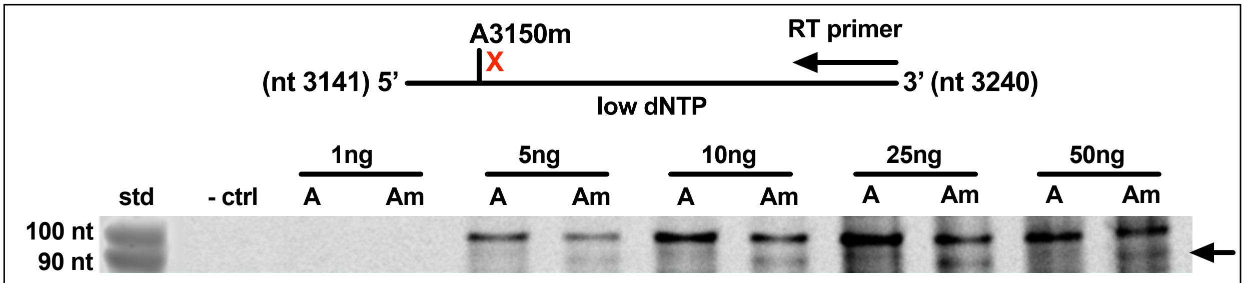

Panel A: Primer extension of *Pxdn* 100-mer RNAs, Nm-modified (Am) and unmodified (A) at position 3150. Amount of template in each reaction is shown in nanograms (ng). The product formed by RT stopping due to Am is at 90 nt (arrow), while the full-length product is 100 nt.

Note that the Am modification not only results in the appropriate stop product (90nt), but also reduces the amount of full-length product. In a mixing experiment of the modified and unmodified templates, we found that the amount of 90nt product is linearly related to the amount of Nm-modified template (**Panel B**). It is critical to note that even with stoichiometric (100%) Nm modification of the A3150 site, there is not 100% stoppage, and the maximum amount of "stop" product generated is only 20-25% of the full length (100-mer) product.

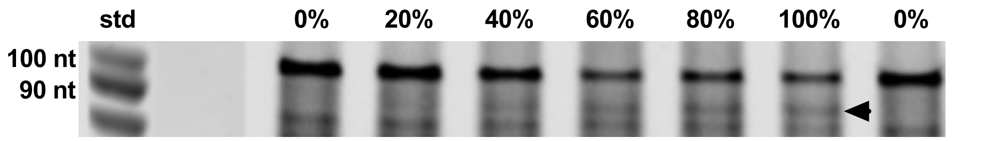

Panel B: Primer extension mixing experiment. %Am is shown for each reaction. RT product due to Am stopping is at 90 nt; full length product is 100 nt. The ratio of 90nt/100nt products (% stop) was plotted against the %Am in each reaction, with an excellent linear relationship.

As noted above, we were never able to obtain any sort of band (not even background "stuttering") when targeting *Pxdn* transcript from total RNA or highly purified mRNA. Based on our synthetic templates above, we needed 5ng of our purified template with 100% Am3150 for it to be visualized on the gel (**Panel A**). For comparison, in a recent study where 2'-*O*-methylation was determined in HIV RNA by primer extension, a full 250 ng of highly purified HIV RNA (a single transcript) was needed to visualize the stops. For our synthetic template, 5ng is approximately 90 billion template copies, which is trivial to achieve with rRNA but perhaps impossible for a single mRNA species. To achieve this in biological samples, knowing the abundance of *Pxdn* is in our cells (determined by ddPCR measurement), we would need to the amount of *Pxdn* mRNA present in at least 375 mg of total RNA (assuming 100% stoichiometric modification and no loss of signal intensity in a complex RNA mixture). At this time, we cannot feasibly collect this much *Pxdn* mRNA from our cells or animals. In the future, we are hopeful that transcript stoichiometry can be determined by RNA-seq methods or perhaps more sensitive methods such as direct LC-MS.

3. It is really interesting to see in Figure 3 that while U32A+U51 KO cells have lower overall protein synthesis, the production of myc-tagged hPXDN is increased. Based on this, the author concluded that the Nm modification inhibit mRNA translation. More experiments are needed to support this conclusion: (i) The amount of myc-tagged hPXDN was normalized to the amount of biotin-labelled protein, while the potential difference in the amount of hPXDN mRNA (for example, caused by the difference in transfection efficiency) was not considered. (ii) To claim that it is the U32A/U51-mediated Nm modification that affects translation, myc-tagged hPXDN constructs with mutations at the proposed 3150Am site (or around it) may need to be tested in the same assay to see if the translation upregulation is abolished or not.

(i) This is an important point as transfection efficiencies can be variable. We have updated our analyses to normalize the nascent PXDN protein signal to both total biotinylated protein and relative *Pxdn* mRNA expression in the WT and KO cells (now **Fig. 4b**). The result of this experiment remains unchanged, as the *U32A+U51* KO cells still show significantly higher PXDN protein synthesis, even when normalized to the mRNA expression levels.

(ii) The suggestion to directly test whether snoRNA-mediated Nm modification of *Pxdn* truly involves the A3150 site is excellent, as all known box C/D snoRNAs have a Watson-Crick base-pair at the site of *Fbl*-mediated Nm modification, and prior work has shown that mutation of the target site inhibits the Nm placement. We therefore mutated the A3150 site to C3150, creating a critical and inhibitory base-pair mismatch with the *U32A* and *U51* snoRNAs (shown in **Fig. 4c)**. When we compared the PXDN protein production from this mutant construct to the native A3150 construct (in WT 293T cells), we found that this single nucleotide point mutation does in fact result in higher levels of *Pxdn* translation (**Fig. 4d**), as would be expected if the site cannot be a target of snoRNA-guided Nm modification. This complements the protein translation results seen in our *U32A+U51* snoRNA KO cells, and provides significant new evidence supporting the hypothesis that A3150 is a target of snoRNA-guided Nm modification.

Minor points:
1. In the abstract, N6-methyladenosine (m6A) was exemplified as a post-transcriptional gene regulator for translation. Wang et al's work on YTHDF1, one of the first mechanistic studies on m6A's function on translation, should be cited as well.

This citation has been added to the text.

2. In Figure 1b, the calculation of each of the Nm level, i.e., the y axis of the figure, should be specified. A "mock" sample with only digestion enzymes but no experimental RNA should be included to account for potential Nm nucleosides from the reagents.

The y-axis in **Fig. 1b** has updated for clarity. As suggested, we also used our UPLC-MS/MS method to analyze three mock nucleoside digestions with no RNA input, and found no detectable RNA nucleosides (modified or unmodified). We have added this information to the text (line 44-45).

Reviewer #3:

1) It does not appear that there is any quantitative analysis of the simulations. Rather, there are only a few snapshots and movies shown, along with some qualitative commentary based on visual inspection of the simulations. While the claims may very well be valid, there is no reason for such a qualitative presentation of the results. Further, it is not clear why the simulations were only performed for 50ns, which is much less than current standards in the field. As the authors are certainly aware, it is no uncommon for systems to require hundreds of nanosecond to properly equilibrate. With modern resources, such a simulation should probably only take about a week on a single compute node (with a GPU). With the relatively low cost of these simulations, it would be worthwhile to significantly extend the duration of the simulations, and perform of systematic analysis of the dynamics (e.g. interatomic distance distributions would be helpful).

We are pleased to report that we have re-run the simulations at a timescale of 500ns (10x longer than before) and also now provide quantitative analysis of the new results (as hydrogen bond donor-acceptor distances, see new **Fig. 5e**). The updated dataset continues to support our original assertion (and other published data), that Nm modification at either the A1 or A2 position of an AAG codon is predicted to be disruptive. The quantitative assessment in **Figure 5e** shows this clearly, where it is easy to see that the modified Am1 nucleoside destabilizes the A1:A1493 interaction. Similarly, the modified Am2 nucleoside is completely incompatible with hydrogen bonding at A2:A1492. We have included a more detailed discussion of these results in the text (lines 155-159) and also extensively revised the figure legend (as suggested below).

2) The caption to figure 4 needs to be substantially improved. As written, there is a lack of scientific content in the caption. Here are a couple examples of how to improve this caption.
- It is not stated how these particular snapshots were chosen. Are they representative, or are they the initial configurations after energy minimization?

- There is a statement "Full simulations available as online movies..." should read "Movies of the full trajectories are available..."
- There is no description provided for the arrows and large green spheres.

We agree that the Figure caption was less than ideal. Building on the Reviewer's suggestions, we have completely re-written the caption for this Figure (which is now **Fig. 5**):

Figure 5 | Molecular dynamics modeling of *Pxdn* Nm codon modification at the ribosomal A-site. SnoRNA-guided Nm modification on *Pxdn* mRNA is predicted at the first position of an AAG lysine codon. Our model of the ribosomal A-site included the mRNA, tRNA, and rRNA, but only the first two nucleotides of the AAG codon (A1 & A2) and the relevant monitoring rRNA nucleotides (A1492 & A1493) are shown for clarity. Hydrogen bonds are shown as yellow lines. Movies of the full trajectories are available online. **a, b**, Representative configurations of the canonical interaction. For the A1:A1493 interaction, note that A1 can serve as the hydrogen bond donor (**a**) or acceptor (**b**), highlighted by arrows. **c, d**, Representative configurations of the Am1 and Am2 modifications, respectively, where the van der Waals radius for the Nm methyl group is shown as a yellow-green sphere. **e**, The A1:A1493 and A2:A1492 donor-acceptor distances are shown for 500ns simulations. Distances < 0.32nm are consistent with hydrogen bonding interactions, and the percent of time that each interaction falls within this range are also shown.

- Is panel e a schematic based entirely on the simulations, or is this a synthesis of all results in the study? If it is a synthesis of the full study, it should be included as a separate figure.
- A full description of panel e should be provided. There are six subpanels, which deserve a full explanation.

The **Fig. 4e** schematic was intended as a synthesis of all results in the study, and we have now moved it to a separate figure (now **Fig. 6**), with a full description in its own Figure Legend (as suggested by the Reviewer):

Figure 6 | Proposed model. Our findings suggest that *Pxdn* mRNA is targeted by snoRNAs for FBL-mediated Nm modification. The presence of this Nm modification in the coding sequence leads to an increase in mRNA abundance but with suppressed translation (left). In the absence of either FBL or the snoRNA guide(s), the mRNA is not Nm-modified, resulting in lower mRNA expression, but the translational suppression is released, leading to paradoxically higher levels of protein expression (right).

References

1. Krogh, N. *et al.* Profiling of 2'-O-Me in human rRNA reveals a subset of fractionally modified positions and provides evidence for ribosome heterogeneity. *Nucleic Acids Res.* **44**, 7884–7895 (2016).
2. Marchand, V., Blanloeil-Oillo, F., Helm, M. & Motorin, Y. Illumina-based RiboMethSeq approach for mapping of 2'-O-Me residues in RNA. *Nucleic Acids Res.* gkw547 (2016). doi:10.1093/nar/gkw547
3. Holley, C. L. *et al.* Cytosolic accumulation of small nucleolar RNAs (snoRNAs) is dynamically regulated by NADPH oxidase. *J. Biol. Chem.* **290**, 11741–8 (2015).
4. Dai, Q. *et al.* Nm-seq maps 2'-O-methylation sites in human mRNA with base precision. *Nat. Methods* (2017). doi:10.1038/nmeth.4294
5. Cheng, G., Salerno, J. C., Cao, Z., Pagano, P. J. & Lambeth, J. D. Identification and characterization of VPO1, a new animal heme-containing peroxidase. *Free Radic. Biol. Med.* **45**, 1682–1694 (2008).
6. Hanmer, K. L. & Mavri-Damelin, D. Peroxidasin is a novel target of the redox-sensitive transcription factor Nrf2. *Gene* **674**, 104–114 (2018).
7. Dong, Z.-W. Z.-W. *et al.* RTL-P: a sensitive approach for detecting sites of 2'-O-methylation in RNA molecules. *Nucleic Acids Res.* **40**, e157 (2012).

REVIEWERS' COMMENTS:

Reviewer #1 (Remarks to the Author):

The authors addressed all points raised from the first round. Just a short comment: in the meantime Nm was also shown to be inhibitory in HEK293T cells (Hoernes et al.; Genes (Basel) 2019).

Reviewer #2 (Remarks to the Author):

The authors have answered my questions.

Reviewer #3 (Remarks to the Author):

The computational aspects of this manuscript were significantly improved upon revision. As noted in the original review, it is not uncommon for equilibration to require more than 100ns. The new simulations serve as an excellent example of this point. By extending the simulations to 500ns, the new trajectories reveal reversible fluctuations that begin to occur after 100-200ns. There are just a few minor questions and suggested changes.:

1) It would be advisable to label all subpanels in figure 5. As written, all simulated trajectories are shown in a single sub-panel (Fig. 5e). This makes is very difficult to follow the text, since all references are to Fig 5e. The remaining points below may result from this issue.

2) The main conclusion from the simulations is that methylation leads to steric exclusion, where there is breaking of mRNA-ribosome interactions. At lines 155-157, there appears to be somewhat contradictory interpretations. Specifically, line 155 reads "is dynamic...." which is followed by "there is a relatively fixed interaction distance." What is meant by a dynamic fixed-distance interaction?

3) It doesn't appear that all of the 5e subpanels are discussed. For example, while Am1 leads to reversible breaking of the A1-A1493 interaction, the opposite is true for the A2-A1492 interaction. That is, the H-bond breaks in wild type (top right panel of 5e), but remains formed in the Am1 mutant. Wouldn't this somewhat contradict the claim that steric occlusion leads to breaking of the mRNA-ribosome interactions? Instead, there may be a compensatory H-bond that is formed, in place of the A1-A1493 interaction. Upon re-reading the text, this may be the point the authors are trying to convey, but the somewhat imprecise description of panels (point 1) may be the reason it is not clear.

NCOMMS-18-16019-A
Point-by-point response to Reviewers
July 3, 2019

Reviewer #1:
The authors addressed all points raised from the first round. Just a short comment: in the meantime Nm was also shown to be inhibitory in HEK293T cells (Hoernes et al.; Genes (Basel) 2019).

Thank you for raising this point. We now reference this publication in our manuscript (Reference #9, lines 40, 141, 171, and 184).

Reviewer #3:
The computational aspects of this manuscript were significantly improved upon revision. As noted in the original review, it is not uncommon for equilibration to require more than 100ns. The new simulations serve as an excellent example of this point. By extending the simulations to 500ns, the new trajectories reveal reversible fluctuations that begin to occur after 100-200ns. There are just a few minor questions and suggested changes.:

1) It would be advisable to label all subpanels in figure 5. As written, all simulated trajectories are shown in a single sub-panel (Fig. 5e). This makes is very difficult to follow the text, since all references are to Fig 5e. The remaining points below may result from this issue.

We have now broken the previous panel of Fig. 5e into six separate subpanels: 5e,f (canonical), g,h (Am1), and i,j (Am2).

2) The main conclusion from the simulations is that methylation leads to steric exclusion, where there is breaking of mRNA-ribosome interactions. At lines 155-157, there appears to be somewhat contradictory interpretations. Specifically, line 155 reads "is dynamic...." which is followed by "there is a relatively fixed interaction distance." What is meant by a dynamic fixed-distance interaction?

(NB: In the revised manuscript, this the Reviewer's comment now refers to lines 174-176.)
As shown in Fig. 5, we are trying to convey that the canonical (unmodified) donor-acceptor relationship between Am1 (mRNA) and A1493 (rRNA) is dynamic, while at the same time maintaining a fixed interaction distance (stable hydrogen bond). The "dynamic" state is that either Am1 can be the H-donor and A1493 can be the acceptor, *or* the 2'-OH groups can rotate, such that A1493 is the H-donor and A1493 is the acceptor. This is seen comparing panels 5a and 5b, and also (dynamically) in the Supplemental Movie 1. Despite the flexibility of this donor-acceptor relationship, the interaction distance remains essentially fixed, as shown in the revised panel 5e (where the interaction distance is <0.32nm for 98% of the time).

3) It doesn't appear that all of the 5e subpanels are discussed. For example, while Am1 leads

to reversible breaking of the A1-A1493 interaction, the opposite is true for the A2-A1492 interaction. That is, the H-bond breaks in wild type (top right panel of 5e), but remains formed in the Am1 mutant. Wouldn't this somewhat contradict the claim that steric occlusion leads to breaking of the mRNA-ribosome interactions? Instead, there may be a compensatory H-bond that is formed, in place of the A1-A1493 interaction. Upon re-reading the text, this may be the point the authors are trying to convey, but the somewhat imprecise description of panels (point 1) may be the reason it is not clear.

We have now revised the text with the detailed sub-panel labeling to clarify this issue (see lines 174-185). Each of the six new subpanels (5e-j) has been referenced in the text. The Reviewer's observation is certainly correct: Our simulations suggest that the Am1 configuration both destabilizes the Am1:A1493 interaction (Fig. 5g vs. 5e) and stabilizes the A2:A1492 hydrogen bond (Fig. 5h vs. 5f). However, we note that this stabilized A2:A1492 bond does not achieve the very high stability of the A1:A1493 bond in the unmodified state (Fig. 5e), so it may not be sufficient "compensation". Most importantly, our biological data suggest that that this stabilization adjacent to the Am1:A1493 disruption is insufficient to prevent an inhibitory effect on translation.
